# Simplified mechanistic models of gene regulation for analysis and design

**DOI:** 10.1098/rsif.2015.0312

**Published:** 2015-07-06

**Authors:** Edward J. Hancock, Guy-Bart Stan, James A. J. Arpino, Antonis Papachristodoulou

**Affiliations:** 1 Department of Engineering Science, University of Oxford, Oxford OX1 3PJ, UK; 2 Department of Bioengineering & Centre for Synthetic Biology and Innovation, Imperial College London, London SW7 2AZ, UK

**Keywords:** gene regulatory networks, systems biology, synthetic biology, mechanistic models, reduced models

## Abstract

Simplified mechanistic models of gene regulation are fundamental to systems biology and essential for synthetic biology. However, conventional simplified models typically have outputs that are not directly measurable and are based on assumptions that do not often hold under experimental conditions. To resolve these issues, we propose a ‘model reduction’ methodology and simplified kinetic models of total mRNA and total protein concentration, which link measurements, models and biochemical mechanisms. The proposed approach is based on assumptions that hold generally and include typical cases in systems and synthetic biology where conventional models do not hold. We use novel assumptions regarding the ‘speed of reactions’, which are required for the methodology to be consistent with experimental data. We also apply the methodology to propose simplified models of gene regulation in the presence of multiple protein binding sites, providing both biological insights and an illustration of the generality of the methodology. Lastly, we show that modelling total protein concentration allows us to address key questions on gene regulation, such as efficiency, burden, competition and modularity.

## 1. Introduction

Gene regulation is fundamental to how both natural and ‘synthetic’ biological systems function, determining everything from how cells respond to environmental changes to differentiation of cell type [1]. Owing to the complexity of gene regulation, model-based approaches are essential for studying all but the simplest genetic networks and simplest observable properties [2–6]. Furthermore, advances in modelling and model-based design are required to overcome a current significant bottleneck in the design and implementation of synthetic gene regulatory networks comprised of more than a few genes. Models of particular importance for both analysis and design are mechanistic models derived from biochemical reactions. These mechanistic models enable DNA sequences and biochemical mechanisms to be related to the observable ‘system’ properties. This direct link from ‘parts’ to ‘systems’ is important for applications, such as for converting a synthetic gene regulation ‘system’ design into the DNA sequences of the ‘parts’ for genetic transfer into a cell.

In practice, these often highly complicated mechanistic models need to be simplified using a ‘model reduction’ approach. Model reduction decreases the number of modelled variables and parameters, often significantly, while retaining the properties and thus advantages of the full mechanistic model. Historically, this approach has been better known for mechanistic modelling of enzymatic reactions rather than for gene regulation, e.g. the extensively studied Michaelis–Menten enzyme kinetics [7]. Model reduction of mechanistic models enables parameter identification from experimental data, which is otherwise a significant challenge [5]. Additionally, reduction also improves computational scalability [3] and enables systems-level analysis and design, including the use of extensive methods for analysing simple empirical models [3,4].

However, conventional ‘reduced’ kinetic models of gene regulation use variables that are not experimentally measured and are based on assumptions that often do not hold under experimental conditions [8,9]. Most systems and synthetic biology studies rely on the quantification of mRNA or protein concentrations through various experimental techniques, e.g. fluorescent reporters [10], microarrays [5] or RNAseq [11]. Typically, these measurement techniques can only reveal total mRNA or protein amounts, such as a transcription factor (TF) in tandem fusion with a fluorescent reporter revealing total TF concentration. These outputs do not match with the single form of TF used in conventional kinetic models, e.g. free monomeric or free multimeric TF concentrations. In this context, modelling either the TF's free monomeric or free multimeric concentration also introduces a large modelling error when the protein is not predominantly in the form of the modelled TF variable [8,9]. Similarly, two forms of TF have been modelled (e.g. total dimer—bound and free) [12,13], with similar restrictive assumptions and measurability issues to previous approaches involving one form of TF. Some progress has been made to find reduced monomeric TF models with ‘corrections’ to account for the error [8,9]. However, these ‘corrected’ models do not have an experimentally measurable output and they use restrictive assumptions based on the ‘speed’ of reactions, which often do not agree with experimental data. Furthermore, these models become highly complex when all required degradation/dilution terms are included. This added complexity limits understanding of system effects, including the use of analysis and design methods in existing literature.

Here, we resolve these issues by proposing a reduction methodology and reduced kinetic models of total mRNA and total protein concentration, which link measurements, models and biochemical mechanisms. The proposed methodology and reduced models are based on assumptions that hold generally and include typical cases in systems and synthetic biology where conventional models do not hold. We propose novel assumptions regarding the ‘speed of reactions’, which are required for the assumptions to be consistent with known experimental data. However, we do not assume that the TF is in a particular form and so remove assumptions that restrict the applicability of conventional models. The direct use of total TF proposed here contrasts with monomeric TF models with ‘corrections’ that use total TF indirectly [8,9,12]. The approach presented here also enables practical applications under experimental conditions by removing the above-mentioned hurdles of measurability, complexity and the use of often unjustified assumptions. In particular, the simplicity and mechanistic accuracy of the models are important for modelling in systems biology while essential for design in synthetic biology.

The different conventional models can be treated as special cases of the proposed approach and so new criteria are provided for cases when the different conventional models may be used or should be avoided. These criteria are based on the reduced parameters of the biochemical models and so are practical to use. The reduced models also use approximated terms, such as the fraction of protein in monomer or dimer form. These approximations can be selected to be as mechanistically accurate as required, and there can be a trade-off between simplicity and accuracy for cases where conventional models do not hold.

We introduce the methodology and simplified models using prototypical cases, noting that the approach can be easily extended to large gene regulatory networks and can be used to incorporate additional mechanistic detail in the simplified models. As such, the approach has wide applicability and can be very informative to a range of networks in systems and synthetic biology. We look at the deterministic case modelled using ordinary differential equations as this is important for simplified analysis and design, and is a widely used first step before analysing the stochastic case. To illustrate the results, we use standard synthetic biology examples for which the proposed models are mechanistically accurate, whereas conventional simplified models produce significant qualitative errors in prediction. We also apply our proposed methodology to derive simplified models of gene regulation in the presence of multiple TF binding sites, providing both biological insights and an illustration of the generality of the methodology. We use the simplified models to analyse an example of a toggle switch, which is bistable only in the presence of additional TF binding sites that do not directly regulate promoter activity. Finally, we show that modelling total protein concentration addresses key questions on gene regulation, such as efficiency, burden, competition, retroactivity and modularity. These concepts are more naturally discussed in terms of total protein, whereas the proposed reduced models allow us to analyse and discuss them in a simplified manner. In particular, we find that adding a downstream module only affects total protein concentration owing to feedback or degradation/dilution rates differing between the bound and unbound forms of TF.

## 2. Results: biochemical model reduction

**Figure 1.**
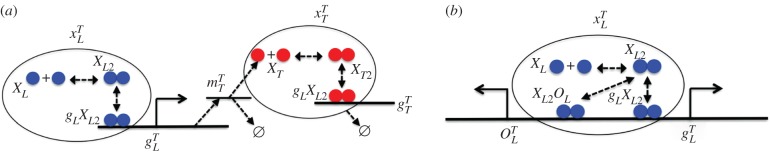
Prototypical genetic network modules. The prototypical input–output system (*a*) with total DNA (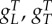), mRNA 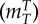 and protein (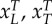) is shown. Dimerization of monomeric input transcription factor (TF) (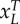) and output TF (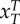) has been considered as well as operator-binding and lumped transcription, translation and degradation. The input–output ‘module’ acts as a building block for modelling larger networks. For the case of multiple operators (*b*), the system also includes the total additional TF binding sites (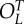) and the total protein concentration also includes the TF bound to the second operator. In (*b*), the additional regulatory element is part of a second promoter, but the models and methodology are also applicable when additional elements regulate expression of the same gene. (Online version in colour.)

To illustrate our framework, we use the simple prototypical gene regulatory network shown in figure 1*a* in which a dimeric TF represses the expression of a second dimeric TF. This case is used to introduce the gene expression models and model reduction methodology, noting that the same methodology and simplified model structure can be used more generally. This generality is demonstrated in the electronic supplementary material and subsequent models. The regulating protein is treated as an input, and the expressed protein as an output. This input–output ‘module’ acts as a building block for larger gene regulatory network models. The prototypical case with added gene regulatory elements (figure 1*b*) is also considered both owing to its importance and to illustrate that the methodology can be used more generally.

### 2.1. Full biochemical model and existing simplified models

The set of biochemical equations for the prototypical gene regulatory network is presented in (2.1). Only the expression and degradation of the expressed protein (output) are included, as the regulating TF (input) is assumed to have equivalent expression and degradation reactions modelled in a separate input–output ‘module’.2.1
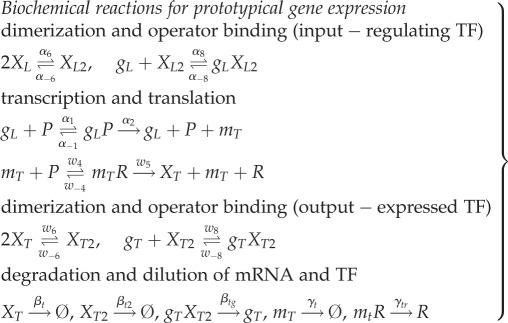


In this model, *g_L_* represents both the promoter driving transcription of mRNA, *m_T_*, and operator-binding sites for the dimeric input TF *X_L_*_2_. Also, *P* is RNA polymerase (RNAP), *R* is ribosome, *X_T_* is the expressed (output) protein monomer, *X_L_* is the regulating (input) free monomer, *X_T_*_2_ is a dimeric TF, *g_T_* is an operator-binding site for *X_T_*_2_ on the output gene, whereas combinations of terms are biochemical complexes. Two *X_L_* monomers can reversibly associate to form *X_L_*_2_ dimers. *X_L_*_2_ dimers can reversibly bind to the operator of the input promoter, which represses transcription of *m_T_* by sequestering the promoter from RNAP. Transcription of *m_T_* is initiated only when RNAP binds to the upstream promoter, *g_L_*, in the absence of bound *X_L_*_2_. Translation occurs when a ribosome, *R*, binds to a ribosome binding site on *m_T_*, which then initiates translation of *X_T_* monomers. Similar to the input, *X_T_* monomers can reversibly associate to form *X_T_*_2_ dimers, which can subsequently bind to an operator sequence, *g_T_*. The biochemical reactions in (2.1) are used to represent the kinetic models using ordinary differential equations derived from the law of mass action [5].

**Figure 2.**
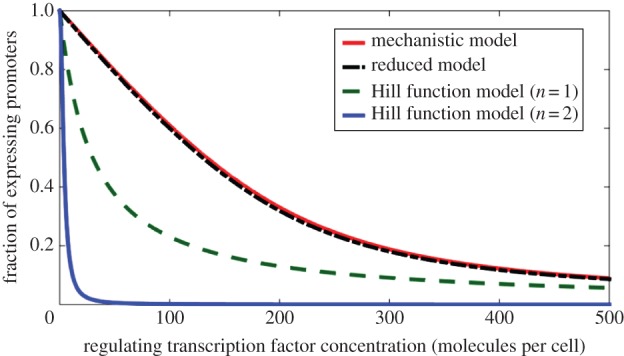
A comparison of protein expression in the full and reduced mechanistic models. There is a close match in protein expression levels between the full mechanistic model and our proposed reduced model, whereas there is an error in the existing reduced Hill function models. (Online version in colour.)

In conventional simplified models, a Hill function is used to represent the relationship between a regulating TF (input) and gene expression from the promoter that it regulates. For empirically derived Hill functions, where the input generically represents the regulating TF, the model's constants and variables cannot be related to the mechanistic model in (2.1), and hence the system behaviour cannot be related to biological parts. For Hill functions obtained from the simplification of mechanistic models [4], where the regulating TF is either the free monomeric TF *X_T_* or the free multimeric TF *X_T_*_2_, the model operates under assumptions that often do not hold, introducing an error [8,9]. Examples of this error can be seen in figure 2.

### 2.2. Reduced biochemical models and multimerization efficiency

We introduce a reduced biochemical model, where the input and output are both total TF concentrations and the model can be used as a building block for larger gene regulatory network models. Using the two concentrations of total mRNA and total protein for each gene, we propose the following reduced biochemical equations:2.2
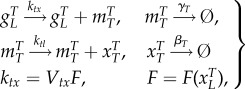
where 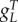 represents the total number of genes, 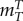 is the total mRNA concentration, 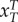 (output) and 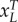 (input) are the total protein concentrations in monomer units, *k_tx_* is the total transcription rate normalized per gene, *k_tl_* is the translation rate per mRNA, *V_tx_* is the transcription rate per non-repressed promoter, *F* is the fraction of promoters that are not repressed and is a function of 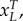 *γ*_*T*_ is the effective mRNA degradation rate and *β*_*T*_ is the effective protein degradation rate (electronic supplementary material, S1–3). The biochemical reactions in (2.2) are used to represent the kinetic models using ordinary differential equations derived from the law of mass action [5]. The parameters in the reduced model (2.2) can be explicitly stated in terms of the kinetic parameters of the mechanistic model (Materials and methods and electronic supplementary material, S1).

We describe gene expression by splitting the model into two separate cases, the choice of which is determined by the biochemical parameters:2.3
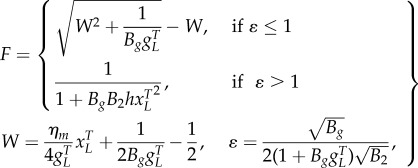
where *B_g_* is the effective dimer–operator association constant for the regulating TF, *B*_2_ is the dimerization association constant for the regulating TF (electronic supplementary material, S1), *η*_*m*_ is the multimerization efficiency, which is the fraction of the regulating TF that is a fully formed multimer, *h* = (1 − *η*_*m*_)^2^ is used to simplify the description, and *W* is used to represent repeated terms in *F* (electronic supplementary material, S2). The two cases are the multimer (*ɛ* ≤ 1) and monomer (*ɛ* > 1) dominant regulation (table 1). If the TF is mostly multimeric when a fraction of the promoters are expressing, then the multimer-dominant case occurs. Conversely, if the TF is mostly monomeric when a fraction of the promoters are expressing, then the monomer-dominant case occurs.

**Table 1.** Biological parameters for transcription factors. Experimental parameter values can be used to determine whether regulation is multimer or monomer dominant in equation (2.3), and when existing models can be used or should be avoided. The monomer-dominant regulation term is used for *ɛ* ≥ 1, whereas the multimer-dominant expression term is used for *ɛ* ≤ 1. For 
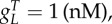
 a mixture of multimer and monomer cases occurs, whereas for 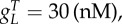 a typical case in synthetic biology [14], only the multimer-dominant case occurs. It should be noted that LacI is a dimer of dimers [4]. Using higher gene copy numbers as an example, tetR may be modelled as only in multimer form (*η*_*m*_ = 1) as *ɛ*_*L*_ ≪ 1, which has previously been used for models fitted to experimental data [12].

transcription factor	1/*B*_2_ (nM)	1/*B_g_* (nM)	*ɛ* for 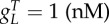	*ɛ* for 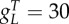
LacI [15–17]	10	10^−2^	0.16	0.0053
TetR [18–21]	1	10	0.14	0.040
AraC [22–24]	10–1000	10	0.45–4.5	0.13–1.3

The multimerization efficiency used to describe expression in the model is estimated, as closely as required, with initial estimates of *h* and *η*_*m*_ in (2.3) of *η*_*m*__0_ = 1 for *ɛ* ≤ 1 or *h*_0_ = 1 for *ɛ* > 1 (electronic supplementary material, S2). Without using estimates, the model is described using more variables in a difficult-to-apply ‘implicit’ form or only described for special cases (electronic supplementary material, S1). The initial approximation is accurate when the system is in a strongly multimer (*ɛ* ≪ 1) or monomer dominant (*ɛ* ≫ 1) case (table 1). For cases where there is roughly an equal mixture of monomeric and multimeric TF (*ɛ* ∼ 1), there is a modelling trade-off between simplicity and accuracy, where multimerization efficiency is a constant for initial approximations, whereas more complicated functions can be used for increased mechanistic accuracy. Using a simple initial approximation followed by a more complicated, but more accurate model allows a step-by-step process of building understanding or completing designs for what can otherwise be difficult-to-analyse models. We can estimate the multimerization efficiency using perturbation theory, where an initial estimate is used to make successively better approximations. Using the initial approximations above, the first iterations of the approximations are2.4
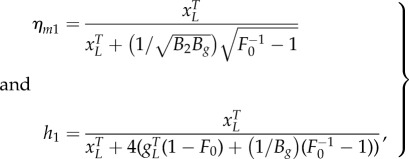
for the multimer and monomer-dominant cases, respectively, which can be used with (2.3) to obtain the first iteration of the regulation function approximation *F*_1_. The error in (2.3) is small for all values of *ɛ* when using the first iteration *F*_1_ (electronic supplementary material, S2). We can alternatively use interpolation to find the approximations of *η*_*m*_ and *h*, where the approximation is ‘calibrated’ for a few particular values of *F* in (2.3). The interpolation approach results in simpler ‘higher-order’ terms, but with an increased error for these approximations (electronic supplementary material, S2). To ensure that (2.4) is well defined, we also need to set *η*_*m*__1_ = 0 and *h*_1_ = 1 for 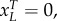 which is only required when 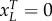 is an initial condition.

If uniform degradation occurs, where different forms of the TF, such as monomer or free multimer have the same degradation rate, we model TF degradation (*β*_*T*_) as a constant. If non-uniform degradation occurs [25], we (closely) approximate the degradation rate as it varies with the output TF concentration 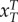 by splitting the model into multiple cases in a similar manner to the regulation term in (2.3) (electronic supplementary material, S3). Uniform degradation is both biologically reasonable in a large number of cases (e.g. the dilution only case) and is a useful first approximation. It should be noted that this definition of uniform degradation does not require two distinct proteins to degrade at the same rate.

We can also model activators (electronic supplementary material, S4), and as is typical in other gene regulation models, only protein concentration is required in the model if the RNA degradation rate is much higher than the protein degradation rate (electronic supplementary material, S5). Furthermore, the models are easily generalizable, where we can include inducers (electronic supplementary material, S7), basal expression (electronic supplementary material, S6), and we can also easily incorporate effects owing to competition for polymerase or ribosomes (electronic supplementary material, S1).

We can compare the full and reduced mechanistic models in terms of their predicted expression levels (as a fraction of the maximum) for varying regulating (input) TF (figure 2). It can be seen that there is a close match in terms of the predicted expression levels between the full and our reduced mechanistic models with different levels of regulating TF. Similarly, it can be seen that our reduced models are qualitatively similar to traditional simplified models, although they can incur a significant quantitative difference. As such, figure 2 also provides examples that show the errors introduced by conventional Hill function models.

### 2.3. Relation to existing models

For the simplest representation of expression in (2.3), the regulation term *F* is a first-order Hill function for the multimer-dominant case and a second-order Hill function for the monomer-dominant case, similar to the forms of traditional models. This can be seen by noting that if the gene concentration is much smaller than the operator binding dissociation constant 
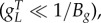
 then we have2.5
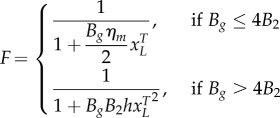
(see electronic supplementary material, S8 for derivation). We note the factor 2 in the denominator for the case *B_g_* ≤ 4*B*_2_ as there are two monomer units in a dimer, and that *h*_1_ in (2.4) also simplifies as the term involving 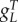 in the denominator can be removed. The repressor regulation in (2.5) reduces to existing simplified models if *η*_*m*_ = 1 or *h* = 1, the initial approximations. The proposed conditions under which these models hold allow us to determine when we can use the different traditional models, and if not, when they may be used as an initial coarse approximation. From this, we can also see that the mechanistic models differ most significantly from traditional models when there is a mixture of TF forms. For this ‘mixed’ case where more complicated expressions for multimerization efficiency are used, the proposed models are related to empirical Hill function models with non-integer orders. Example of the modelling error of conventional Hill function for these mixed cases can be seen in figure 2. Both (2.5) and a Hill function model with a non-integer order contain the same number of variables and parameters. However, when operator occupancy is important as in (2.3), the reduced models require an extra parameter (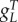) to describe regulation.

Interestingly, gene concentration is often high in synthetic biology experiments, as the artificial genetic material for *in vivo* prokaryotic implementation is often encoded on plasmids, which can be at much higher numbers per cell than chromosomally integrated genes [14]. Thus, the proposed model for multimer-dominant regulation in (2.3) is essential for synthetic biology, but also highly useful for systems biology, where the assumption regarding gene copy number may not hold.

The proposed reduced kinetic models can also be contrasted with complementary thermodynamic equilibrium models, which have also used total TF as a variable [26–30]. Thermodynamic equilibrium models are complementary to kinetic models as they can relate parameters to genetic sequences [31]. We describe gene regulation in a simpler explicit form, which removes the need for difficult-to-apply implicit forms containing more variables or the restrictive assumptions commonly used in equilibrium models. As will be shown in §2.4, the combined use of kinetic and equilibrium models has also been enhanced by deriving conditions under which equilibrium models are valid for use in combination with kinetic models.

### 2.4. Assumptions: speed of reactions

We find that the proposed reduced models are a close approximation of the full mechanistic model when the degradation rates are the time-limiting steps in the biochemical network, the typical case (electronic supplementary material, S1). By this, we mean that the lifetimes of the proteins and mRNA, determined by the degradation rates, provide the ‘natural’ time scale of the dynamics, and that the degradation rates are much ‘slower’ than multimerization, operator-binding, transcription and translation rates [4,32] (electronic supplementary material, S1). This is important to state, as a common unjustified assumption made for ‘quasi-steady state’ reduced models is that the binding rates have to be faster than the transcription and translation rates. The assumption that the degradation rates are the time-limiting steps can be quantitatively written2.6
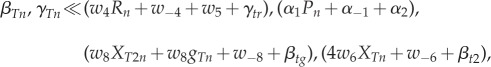
where *R_n_*, *P_n_*, *X_Tn_*, *X_T2n_*, *g_Tn_* are the typical maximum concentrations of the biochemical species, *β*_*Tn*_ is the effective protein degradation at the typical maximum total TF concentration 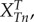 and *γ*_*Tn*_ is the typical maximum of the effective mRNA degradation rate (electronic supplementary material, S1). If required, the typical maximum concentrations can be calculated from the kinetic rates (electronic supplementary material, S1). Equivalent assumptions to (2.6) can be stated for the regulating protein and other transcription/translation reactions. We also require further assumptions to ensure that the time scales of the various fast reactions are not strongly coupled (electronic supplementary material, S1), which typically hold when (2.6) holds. The reduced model ‘loses’ information about the ‘fast’ dynamics owing to the ‘time-limiting’ assumption, but this time scale is not typically relevant for experiments and can be modelled separately if required. In cases where the time-scale separation assumption only holds weakly then the reduced model still provides a ‘coarse’ approximation. The reduction step from an implicit to explicit model can also result in a ‘coarse’ approximation, but only if simplicity is selected over mechanistic accuracy in the multimerization efficiency approximation.

The novel ‘time-limiting’ assumptions generalize those in existing literature, and are required for the methodology to be consistent with known experimental data. The process of transcription and translation initiation is typically much faster than degradation [32,33]. However, validating the assumptions regarding multimerization and operator binding experimentally is not easy. The reverse rate of TF binding has previously been used to determine the speed of the ‘fast’ reaction in monomeric TF models with ‘correction factors’ [8,9]. However, for the example of LacI, the reverse rate of operator binding (time scale of 5–10 min [32]) is often slower than mRNA degradation (approx. 5 min [4]), and is not significantly faster than the full range of protein degradation/dilution rates. In this case, only the speed of the forward rate of operator binding (approx. 30 s [32]) is much faster than mRNA and protein degradation. Unlike previous methods, the assumptions proposed here hold if the forward or the reverse rate are much faster, consistent with experimental data. The methodology also generalizes the number of biochemical reactions to be taken into account when analysing time-scale separation.

### 2.5. Examples: the toggle switch and the repressilator

**Figure 3.**
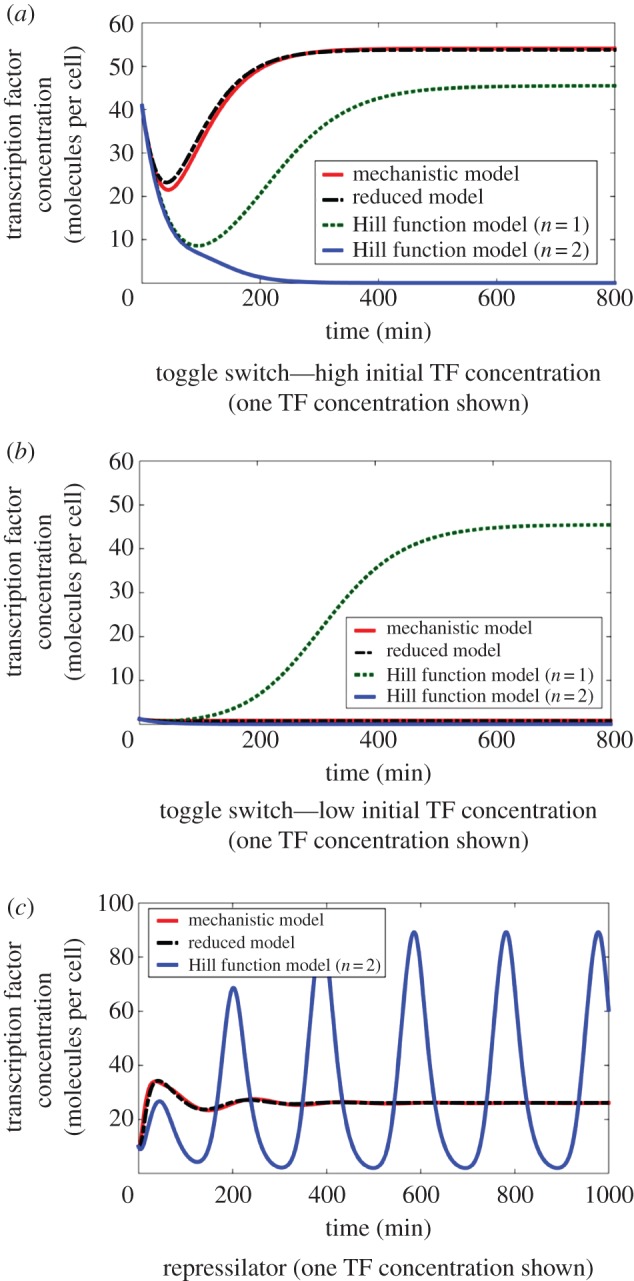
Simulation comparisons of the full and reduced mechanistic models for the toggle switch (*a,b*) and repressilator (*c*). The simulations show that our reduced model matches closely to the full mechanistic model for both simulated networks whilst the Hill function models present both quantitative and qualitative errors. A close match of the reduced models with the detailed mechanistic models is required in order to relate biological parts to systems behaviour. (Online version in colour.)

We demonstrate the application and mechanistic accuracy of our reduced model by comparing simulations of the full and reduced mechanistic models, along with cases that show the errors introduced by Hill function models. A close match of a reduced model with a detailed mechanistic model is required in order to relate DNA sequences and biological parts to systems behaviour for analysis and design. We compare simulations (figure 3) of the toggle switch [34] and the repressilator [35], two standard genetic circuits in synthetic biology.

The proposed model for a genetic toggle switch (figure 3*a*,*b* and Materials and methods) is created by connecting two repressor modules together, where each TF represses expression of the other TF [34] (see figure 1*a* for one module). For the simulations of the reduced models, the reduced parameters in (2.2) and (2.3) are determined by the individual kinetic rates of a full mechanistic model (see Materials and methods for parameter values and equations). Calculating reduced parameters in this way is carried out to compare the full mechanistic model with the proposed reduced models. However, when using the reduced model with experimental data, the parameters in the reduced models can be determined directly, while still allowing predictions of the effects of changes in individual kinetic rates. The value of parameter *ɛ* is next calculated using (2.3) for each TF to select between the multimer- (*ɛ* ≤ 1) and monomer-dominant (*ɛ* ≥ 1) cases of *F* in (2.3). For the toggle switch simulated in figure 3, one TF is dimer-dominant (*ɛ* = 0.14 ≤ 1), whereas the second TF is weakly monomer-dominant (*ɛ* = 1.35 ≥ 1). First-order approximations of *η*_*m*_ and *h* were used as described in (2.4), although using *η*_*m*_ = 1 to model the effect of the TF with *ɛ* = 0.14 ≪ 1 is also a reasonable approximation. In the simulations, degradation is assumed to be uniform, and the free polymerase and ribosome concentrations are assumed constant for simplicity, although these assumptions are not necessary for the methodology to be applied.

The proposed model for a repressilator (figure 3*c* and Materials and methods) is produced by connecting three repressor modules together in a loop [35]. The process of creating the reduced model for the repressilator is similar to the toggle switch. For the repressilator modelled here, there is weakly dimer-dominant regulation for all three TFs (*ɛ* = 0.78 ≤ 1).

We can see a close match between our reduced model and the full mechanistic model for the genetic toggle switch (figure 3*a,b*) and the repressilator (figure 3*c*), whereas the Hill function models introduce a significant qualitative error. This close match between the mechanistic and reduced model shows that we have retained mechanistic accuracy in our reduced model. We can also see that the predictions of the Hill function models do not match with the mechanistic model for both the repressilator and toggle switch. Furthermore, the Hill function models predict the wrong qualitative systems behaviour given the parameters for the biological parts, incorrectly predicting oscillations in the repressilator and predicting no memory in the toggle switch. For the toggle switch, the two Hill function models even predict different qualitative behaviour from each other, with the second-order model predicting an ‘always off’ switch, whereas the first-order model predicts an ‘always on’ switch.

The reduced simplified models have the advantage of requiring fewer biological parameters than the full mechanistic model to complete *in silico* analysis. For example, only the effective dimer–operator association constant is required instead of the individual operator binding and unbinding kinetic rates. This is crucial for *in silico* analysis as it is typically difficult or even impossible to obtain values of individual kinetic rates. The proposed methodology and simplified models also allow simplified analysis compared with the full mechanistic model.

Therefore, our proposed methodology and models have the advantages of mechanistic accuracy compared with conventional reduced models, while allowing practical *in silico* analysis when compared with the full mechanistic models.

### 2.6. Multiple gene regulatory elements

We also apply the methodology to mechanistic models that allow TF to bind to DNA at sites other than the primary operator (figure 1*b*). Modelling multiple TF binding sites is important for understanding how the different operators that bind the same TF are indirectly coupled, both when the operators affect the same and different promoters. It is also crucial for understanding generic effects, such as non-specific binding. Additional ‘sequestering’ gene regulatory elements can be included in the model to determine the effect on the primary operator (electronic supplementary material, S9). The added regulatory element can be modelled using the biochemical reactions2.7
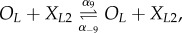
where *O_L_* represents the number of free binding sites owing to added regulatory elements.

For monomer-dominant regulation, there is typically only a small effect from added operators (electronic supplementary material, S9). For multimer-dominant regulation, we split the model into three separate cases, where the binding affinity of the added operator is higher than, approximately equal to or lower than the original binding affinity. Adding higher affinity operators effectively decreases the total protein (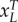) ‘seen’ by the primary operator in (2.3) by sequestering a fraction of the TF; adding approximately equal affinity operators effectively increases the gene copy number (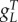) in the regulation term in (2.3) (fraction of promoters expressing), but not in (2.2) (total promoters); while adding lower affinity operators effectively weakens the binding affinity (*B_g_*) of the primary operator (2.3) (electronic supplementary material, S9). These sequestering effects have more impact for higher gene copy numbers and higher operator-binding association constants. The modification to (2.3) owing to added binding sites can be described quantitatively as2.8
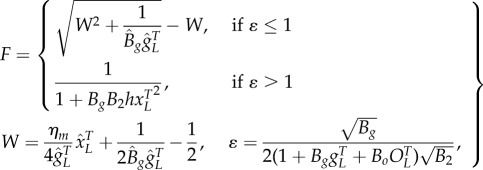
where *B_o_* is the effective dimer–operator association constant of the TF to the additional binding site, 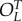 represents the total number of binding sites owing to added regulatory elements and 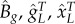 are the modified effective values of 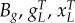 in (2.8), and are described by2.9
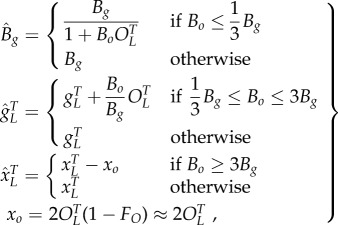
where *x_o_* estimates the concentration of TF (in monomer units) bound to *O_L_*, *F_O_* is evaluated using *F* in (2.8), modified by swapping parameters 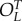 with 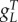 and *B_o_* with *B_g_*, and 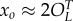 is a coarse approximation when 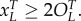 The values of 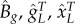 are determined in a similar manner to the multimerization efficiency or non-uniform degradation described above (electronic supplementary material, S9). The initial approximation is shown in (2.9), whereas higher-order approximation can also be used (electronic supplementary material, S9). Also, the higher-order approximation of *η*_*m*_ is unchanged for the multimer-dominant case, but changes for the monomer-dominant case (electronic supplementary material, S9).

### 2.7. An example: the toggle switch with competitive transcription factor binding sites

**Figure 4.**
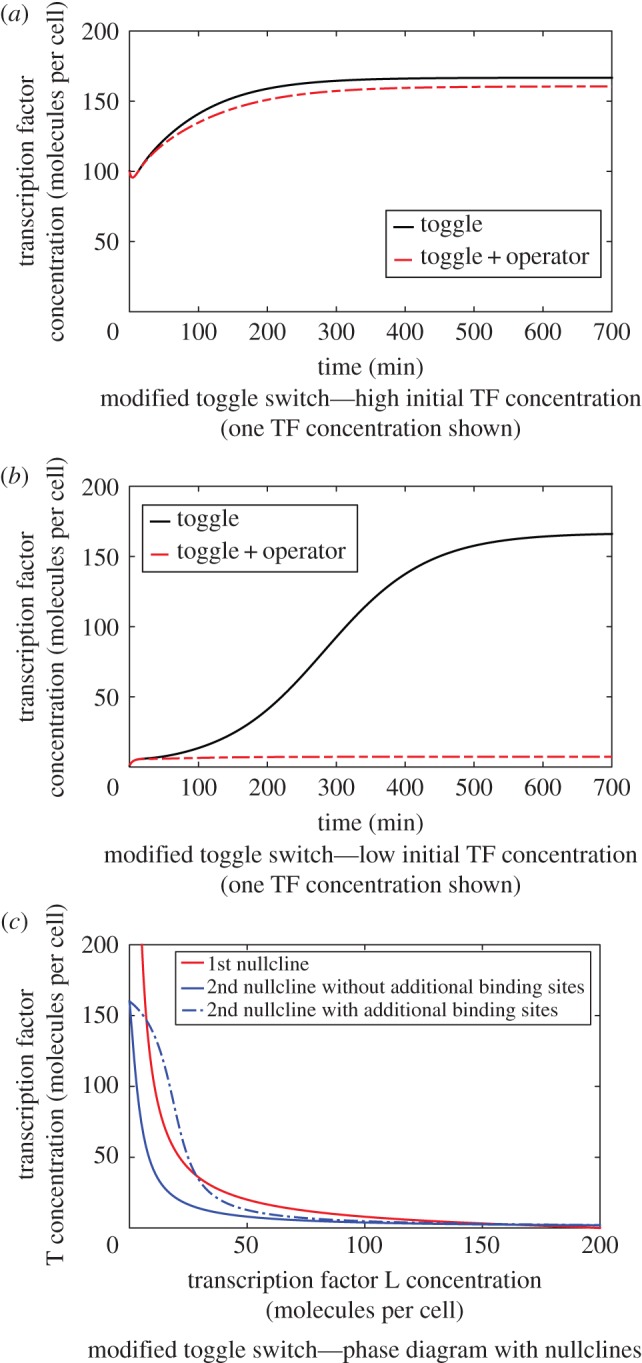
Simulations (*a*,*b*) and phase plane analysis (*c*) for a toggle switch with and without additional TF binding sites. The simulations show that the additional TF binding site can cause bistability in the toggle switch. (Online version in colour.)

In this case study, we demonstrate the simplified *in silico* analysis of models with additional biochemical mechanisms through simulations and graphical analysis of a toggle switch (figure 4 and Materials and methods). We model a toggle switch with and without an additional ‘competitive’ TF binding site for one of the TFs. The approach used to produce a simplified model for this case is similar to the approach used to produce the model without additional TF binding sites described above. However, in this case, we determine both *ɛ* in (2.8), as well as the relative values of *B_g_* and *B_o_*. In the case simulated in figure 4, the regulation is dimer dominant for both TFs (*ɛ* = 0.32, 0.34 without and *ɛ* = 0.32, 0.01 with additional sites) and the dimer–operator association constant is much higher for the additional binding site (*B*_*o*_ ≫ *B*_*g*_). The relationship *B_o_* ≥ 3*B_g_* implies that effective total protein 
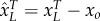
 in (2.9) is reduced, whereas *B̂*_*g*_ = *B*_*g*_ and 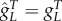 remain unchanged. The significantly higher dimer–operator association constant (*B*_*o*_ ≫ *B*_*g*_) allows the initial approximation in (2.9) to be used. In contrast, if for example *B_o_* ≈ 3*B_g_*, then higher-order approximations of *x_o_* in (2.9) would typically be required (electronic supplementary material, S9).

The simulations and phase plane (figure 4) show that without the additional binding site the toggle switch is monostable, whereas the additional TF binding site can cause bistability in the toggle switch. For this case, there is once again a close match between the full and reduced mechanistic models (electronic supplementary material, S9). This example shows the importance of the methodology and simplified models for cases with multiple TF binding sites. Here, we complete our *in silico* analysis with additional mechanisms without the need to know all biochemical kinetic rates of the full mechanistic model. The reduced model is also particularly suited for exhibiting the effects of these additional mechanisms using phase plane graphical analysis (figure 4*c*), as well as generally for simplified analysis.

## 3. Discussion

We have presented a new model reduction methodology and reduced models of total mRNA and total protein concentrations, which can be directly related to both experimental outputs and the underlying biochemical mechanisms. As different mechanistic models can easily be incorporated into our reduced models using the developed methodology and the models can easily be extended to large networks, we now discuss questions that are relevant for a range of mechanistic models and gene regulatory networks. Using the proposed reduced models, we find that we can gain key insights into gene regulatory efficiency, burden, competition and modularity by modelling total protein and mRNA, while the reduced models enable a simplified analysis.

### 3.1. Regulatory efficiency

An efficient gene regulatory network can potentially reduce burden on the cell or allow a faster response [27]. Therefore, we start with the characterization of an efficient TF, given that regulatory efficiency is an important concept in gene regulation. Using the model in (2.1), we define the efficiency of the TF to be the fraction of the total TF concentration, in monomer units, which is bound to the operator (electronic supplementary material, S10), as this is the only form of the protein in the prototypical example which has a functional effect on gene regulation. We can also estimate the efficiency of regulation using (2.3) (electronic supplementary material, S10). A completely efficient TF is one in which all molecules are bound to an operator until all operators are occupied. For gene regulation, neither free monomers nor free dimers have a functional effect, and so the operator-bound protein can be viewed as an alternative output variable to the total protein concentration. In terms of efficiency, the total concentration of free monomeric and free multimeric TF can be viewed as a measure of the inefficiency of the system. Interestingly, regulatory efficiency is important both by itself, and in determining trade-offs with robustness [36,37]. For example, a concentration drop in a highly efficient repressor can unnecessarily turn on gene expression while an inefficient repressor may act as a buffer.

### 3.2. Loading and retroactivity

Another important question for both synthetic and natural systems is to determine the effect of connecting a single ‘downstream module’ on an ‘upstream module’. This question can be framed in terms of loading and retroactivity [9,12,38–40], where retroactivity describes the connection of a ‘downstream’ network module affecting the ‘upstream’ module's output, which in previous studies has been the free monomeric TF [9].

However, when using the total protein concentration as the module output, adding a downstream operator does not introduce retroactivity unless there is either non-uniform degradation or feedback. This can be seen in the prototypical example with uniform degradation (electronic supplementary material, S3), where the addition of an operator binding to the output TF has no effect on its dynamics, assuming that the output TF does not affect the regulating TF through feedback. Thus, retroactivity is a system property that is dependent upon the choice of output, which in our case is the experimentally measurable output. This dependence on the choice of output has also been seen for stochastic effects [40]. Interestingly, using the total protein concentration allows a simplified identification and analysis of module interconnections when dilution is dominant over degradation and no feedback occurs.

When there are multiple genes regulated by the same TF (figure 1*b*), then the different operators ‘compete’ for the available TF. This ‘parallel loading’ can be predicted by our reduced models with sequestering operators as described above (electronic supplementary material, S9), noting that the effect is typically much stronger for multimer-dominant regulation. In fact, a TF with a higher regulatory efficiency (electronic supplementary material, S10) will typically have a larger parallel loading effect, a loading/efficiency trade-off. The case of multiple genes regulated by one TF has been examined experimentally and with a mechanistic model [12]. Here, we can analyse a TF regulating multiple genes with more mechanisms using a simpler framework and an experimentally measurable output. Similar to the single operator case, parallel loading does not cause a retroactivity effect without either feedback or non-uniform degradation. For this case, the competition between operators only affects the operator-bound concentrations, and does not affect the total protein concentration ‘output’. If one of the competing operators is part of a feedback mechanism, then parallel loading does become a type of retroactivity, or more generally, a network loading effect.

## 4. Conclusion

We have presented a new model reduction methodology and the resulting simplified mechanistic models using total mRNA and total protein concentrations as variables, which link the simplified models with experimental outputs and the underlying biochemical mechanisms. The proposed methodology and models have allowed us to overcome important challenges in using conventional simplified models for applications in systems and synthetic biology. The proposed methodology and models use assumptions that hold generally, and also provide new criteria for when the different conventional models may be used or should be avoided. We provided biological examples where proposed models are mechanistically accurate, whereas the conventional models make significant qualitative errors in prediction. We also applied the methodology to propose simplified models of gene regulation in the presence of multiple TF binding sites. Finally, describing gene regulation using the total protein concentration led to a number of enlightening interpretations, such as regulatory efficiency, while using the proposed reduced model allows for simplified analysis and design of gene regulatory networks.

## 5. Material and methods

Simulation and calculations were completed using Matlab. The function ODE45 was used to simulate reduced ODE models, whereas the function ODE15s was used to simulate reduced differential-algebraic equation (DAE) models (electronic supplementary material, S1) and full mechanistic models.

The reduced parameters are related to kinetic parameters in the full mechanistic models using5.1
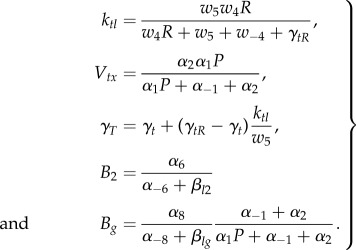
The parameter values used to generate the simulation results presented in figure 2 are 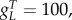 1/*B_g_* = 15, 1/*B*_2_ = 1 (molecules per cell).

The parameter values used to generate the simulation results presented in figure 3*a*,*b* are *P* = 100, *R* = 100, *a*_4_ = 0.1, *a*_−4_ = 1, *a*_5_ = 1, *a*_6_ = 0.01, *a_m_*_6_ = 2, *a*_8_ = 1, *a*_−8_ = 0.001, *a*_1_ = 0.01, *a*_−1_ = 1, *a*_2_ = 1, *β*_*t*__1_ = *β*_*t*__2_ = *β*_*tg*_ = *β*_*l*__1_ = *β*_*l*__2_ = *β*_*lg*_ = 0.025, *γ*_*tr*_ = *γ*_*t*_ = *γ*_*tr*_ = *γ*_*l*_ = 0.2, *w*_4_ = 0.02, *w*_−4_ = 0.2, *w*_5_ = 0.2, *w*_6_ = 1, *w*_−6_ = 2, *w*_8_ = 1.2, *w*_−8_ = 0.01, *w*_1_ = 0.01, *w*_−1_ = 1, *w*_2_ = 1, where the two TFs are *X_T_* and *X_L_*. The initial conditions are chosen as 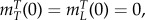 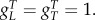 The two initial conditions (high and low) are chosen as *X_L_*_1_(0) = 30, *X_T_*_1_(0) = 1 (high) and *X_L_*_1_(0) = 1, *X_T_*_1_(0) = 3 (low) with other initial conditions set at quasi-steady state (electronic supplementary material, S1). 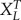 is plotted in figure 3.

The parameter values used to generate the simulation results presented in figure 3*c* are *P* = 1000, *R* = 1000, *a*_4_ = 0.01, *a*_−4_ = 1, *a*_5_ = 1, *a*_6_ = 0.1, *a*_−6_ = 1, *a*_8_ = 0.5, *a*_−8_ = 0.1, *a*_1_ = 0.01, *a*_−1_ = 1, *a*_2_ = 1, *β*_*L*__1_ = 0.05, *β*_*L*__2_ = 0.05, *β*_*Lg*_ = 0.05, γ*_LR_* = 0.1, γ*_Lu_* = 0.1, with identical parameters for all three genes. The initial conditions are chosen as 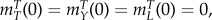 *X_L_*_1_(0) = *X_T_*_1_(0) = 10, *X_Y_*_1_(0) = 20, 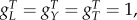 and other initial conditions set at quasi-steady state (electronic supplementary material, S1).

The parameter values used to generate the simulation results presented in figure 4 are 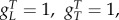 *V_tx,T_* = 1, *V_tx,L_* = 1, *k_tl,T_* = 0.8, *k_tl,L_* = 1, *γ*_*L*_ = *γ*_*T*_ = 0.2, *B_Lg_* = 0.9, *B_Tg_* = 0.4, *B_L_*_2_ = 0.5, *B_T_*_2_ = 0.5, *β*_*T*_ = 0.025, *β*_*L*_ = 0.025. The perturbed system uses the additional parameter values 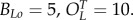 The initial conditions are chosen as 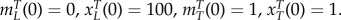


## Supplementary Material

Supplementary Information
