# Supplementary material for: Simplified mechanistic models of gene regulation for analysis and design
Source: J R Soc Interface. 2015 Jul 6;12(108):20150312. doi: 10.1098/rsif.2015.0312 (PMC4528597; doi:10.1098/rsif.2015.0312)
Supplement: Supplementary Information [file rsif20150312supp1.pdf]

Simplified Mechanistic Models of Gene Regulation for Analysis and Design: Supplementary Information

Edward J. Hancock, Guy-Bart Stan, James A.J Arpino and Antonis Papachristodoulou

1 Reduction of Mechanistic Models using Singular Perturbation

In this section, we derive a low order model of total mRNA and protein concentration with general assumptions, by rigorously applying singular perturbation theory [9, 10] to gene regulation models. We obtain a 'reduced' model in the form of a Differential-Algebraic Equation (DAE), where the terms for transcription and protein degradation are described implicitly, noting that we present methodology to describe them explicitly in SI 2 and 3. We first present the prototypical mechanistic model as an Ordinary Differential Equation (ODE) and the proposed reduced model as a DAE. We then prove that the model reduction holds by using a transformed mechanistic model and a novel non-dimensionalisation to obtain a mechanistic model in standard singular perturbation form.

The proposed methodology allows more general assumptions than previous methods that used total TF (Transcription Factor) indirectly [8, 6], necessary to match with known experimental data. This methodology also allows reduction of more complicated mechanistic models than existing methods, allowing the inclusion of qualitatively important biochemical mechanisms previously ignored for tractability.

The model reduction in this section is analogous to the derivation of Michaelis-Menten kinetics [10], particularly derivations using a change of variable or 'total' quasi-steady state [3], noting that here we transform both 'slow' and 'fast' variables.

Mechanistic Model

We first state the ODEs for the full mechanistic model in Equation 1 in the paper. We model the output protein X_T and associate forms $X_{T2}, g_T X_{T2}$, while we treat the protein X_L and associated forms $X_{L2}, g_L X_{L2}$ as an input. When modelling the concentrations, we define the variables $C_{1T} = [g_T P]$, $C_{2T} = [g_T X_{T2}]$, $C_{3T} = [m_T R]$. We assume mass-action kinetics to derive the ODEs, noting that we assume that the dynamics of X_L are equivalent to X_T, and similarly

with other protein concentrations. The full ODE model is

$$
\begin{aligned}
\dot{m}_T &= -w_4 m_T R + (w_{-4} + w_5) C_{3T} + \alpha_2 C_{1L} - \gamma_t m_T \\
\dot{C}_{3T} &= w_4 m_T R - (w_{-4} + w_5 + \gamma_{tR}) C_{3L} \\
\dot{X}_T &= -2 w_6 X_T^2 + 2 w_{-6} X_{T2} + w_5 C_{3T} - \beta_t T \\
\dot{X}_{T2} &= w_6 X_T^2 - w_{-6} X_{T2} - w_8 g_T X_{T2} + w_{-8} C_{2T} - \beta_{t2} X_{T2} \\
\dot{g}_T &= -w_1 P g_T + (w_{-1} + w_2) C_{1T} - w_8 g_T X_{T2} + w_{-8} C_{2T} + \beta_{gt} C_{2T} \\
\dot{C}_{1T} &= w_1 P g_T - (w_{-1} + w_2) C_{1T} \\
\dot{C}_{2T} &= w_8 g_T X_{T2} - w_{-8} C_{2T} - \beta_{tg} C_{2T}
\end{aligned} \tag{1}
$$

where the definitions of kinetic rates and species can be found in the paper (Equation 1).

The Reduced Model and Quasi-Steady State Approximation

We next derive the reduced model using the quasi-steady state approximation. To complete this, we set the summed variables

$$
\begin{aligned}
m_T^T &= m_T + C_{3T} \\
x_T^T &= X_T + 2X_{T2} + 2C_{2T} \\
z_T^T &= 2X_{T2} + 2C_{2T} \\
x_L^T &= X_L + 2X_{L2} + 2C_{2L}
\end{aligned} \tag{2}
$$

which equate to the total mRNA concentration, the total protein concentration, and the total dimer concentration, in both bounds and unbound form. Using the variables z_T^T together with x_T^T simplifies the application of singular perturbation theory to the mechanistic model, as we will see below.

We find the reduced model with the proposed slow variables of total mRNA m_T^T and total protein x_T^T, while all other variables are assumed fast variables. The proposed slow variables have dynamics

$$
\begin{aligned}
\dot{m}_T^T &= \alpha_2 C_{1L} - \gamma_t m_T^T - (\gamma_{tR} - \gamma_t) C_{3T} \\
\dot{x}_T^T &= w_5 C_{3T} - \beta_t x_T^T - (\beta_{t2} - \beta_t) z_T^T - 2(\beta_{tg} - \beta_{t2}) C_{2T}
\end{aligned} \tag{3}
$$

and the quasi-steady state approximations together with the conserved quantities are

$$
\begin{aligned}
g_L + C_{1L} + C_{2L} &= g_L^T \\
\alpha_6 X_L^2 &= (\alpha_{-6} + \beta_{l2}) X_{L2} \\
\alpha_8 g_L X_{L2} &= (\alpha_{-8} + \beta_{lg}) C_{2L} \\
\alpha_1 P g_L &= (\alpha_{-1} + \alpha_2) C_{1L} \\
w_4 R m_T^T &= (w_4 R + w_{-4} + w_5 + \gamma_{tR}) C_{3T} \\
g_T + C_{1T} + C_{2T} &= g_T^T \\
w_6 X_T^2 &= (w_{-6} + \beta_{l2}) X_{T2} \\
w_8 g_T X_{T2} &= (w_{-8} + \beta_{tg}) C_{2T} \\
w_1 P g_T &= (w_{-1} + w_2) C_{1T}
\end{aligned} \tag{4}
$$

We include the degradation/dilution terms $\beta_{l2} X_{L2}$ for operator dissociation and $\beta_{lg} C_{2L}$ for dimer dissociation as this more closely matches experimental measurements for dissociation

[7] and reduces the error. We also include $\gamma_t R$ in the ribosome binding quais-steady state for consistency.

Equation (1) has multiple conserved quantities, including $g_T + C_{1T} + C_{2T} = g_T^T$ shown above as well as

$$\begin{aligned} R + C_{3T} &= R_T \\ P + C_{1T} &= P_T \end{aligned} \tag{5}$$

However, the Ribosome and Polymerase are used by other genes, and so reduced models in terms of R_T and P_T in this form are specific to the prototype gene regulation, whereas we wish to develop a more general methodology. Thus, we treat $R = R(t)$ and $P = P(t)$ as time-varying parameters. This allows us to easily substitute terms involving all bound forms of RNAp and ribosomes when modelling gene regulatory networks instead of a single input-output 'module' presented here. As such, we can model the effects from competition of polymerase and ribosomes.

We next use the variables C_{2T} and C_{2L} to reduce the equations, as they are directly related to expression levels in the model and so act as a proxy. We have the reduced differential-algebraic equations

$$\begin{aligned} \dot{m}_T^T &= g_L^T V_T^{tx} F_L - \gamma_T m_T^T \\ \dot{x}_T^T &= k_T^{tl} m_T^T - \beta_T x_T^T \\ F_L &= 1 - \frac{C_{2L}}{g_L^T} \\ \beta_T &= \beta_t + (\beta_{t2} - \beta_t)\frac{z_T^T(C_{2T})}{x_T^T} + (\beta_{tg} - \beta_{t2})\frac{2C_{2T}}{x_T^T} \\ &= \beta_t + (\beta_{t2} - \beta_t)\frac{2C_{2T}}{x_T^T B_{Tg}(g_L^T - C_{2T})} + (\beta_{tg} - \beta_t)\frac{2C_{2T}}{x_T^T} \\ x_L^T &= \theta_{L1}(C_{2L}) := \frac{\sqrt{C_{2L}}}{\sqrt{B_{L2}B_{Lg}(g_L^T - C_{2L})}} + \frac{2C_{2L}}{B_{Lg}(g_L^T - C_{2L})} + 2C_{2L} \\ x_T^T &= \theta_{T1}(C_{2T}) := \frac{\sqrt{C_{2T}}}{\sqrt{B_{T2}B_{Tg}(g_T^T - C_{2T})}} + \frac{2C_{2T}}{B_{Tg}(g_L^T - C_{2T})} + 2C_{2T} \end{aligned} \tag{6}$$

and we have the reduced parameters

$$\begin{aligned} k_T^{tl} &= w_5 A_{2T}, \quad V_T^{tx} = \alpha_2 A_{1L} \\ B_{Lg} &= \frac{\alpha_8}{\alpha_{-8} + \beta_{lg}}(1 - A_{1L}), \quad B_{L2} = \frac{\alpha_6}{\alpha_{-6} + \beta_{l2}} \\ B_{Tg} &= \frac{w_8}{w_{-8} + \beta_{tg}}(1 - A_{1T}), \quad B_{T2} = \frac{w_6}{w_{-6} + \beta_{t2}} \\ \gamma_T &= \gamma_t + (\gamma_{tR} - \gamma_t)A_{2T} \end{aligned} \tag{7}$$

such that V_T^{tx} is the transcription rate per non-repressed promoter, F_L is the fraction of non-repressed promoters, B_{L2}, B_{T2} are the effective dimerisation association constants,B_{Lg}, B_{Lg} are the effective dimer-operator association constants, γ_T, β_T are the effective mRNA and protein degradation rates, A_{1L}, A_{1T} are the fraction of expressing promoters bound by polymerase,

and A_{2T} is the fraction of RBS (Ribosome Binding Sites) bound by ribosomes, such that

$$\begin{aligned} A_{1L} &= \frac{w_1 P}{w_{-1} + w_1 P + w_2}, \quad A_{1T} = \frac{w_1 P}{w_1 P + w_{-1} + w_2} \\ A_{2T} &= \frac{w_4 R}{w_{-4} + w_5 + w_4 R + \gamma_{tR}} \end{aligned} \tag{8}$$

The parameters A_{1L}, A_{1T}, A_{1T} are used to simplify terms, and are not reduced parameters or kinetic rates.

We can further simplify the parameters $B_{L2}, B_{T2}, B_{Lg}, B_{Lg}$ and A_{2T} by removing the degradation terms if they are sufficiently small ($w_{-8} \gg \beta_{tg}$, $w_{-6} \gg \beta_{t2}$ or $w_4 P + w_{-4} + w_5 \gg \beta_{tg}$). The effect of polymerase binding on B_{Lg}, B_{Tg} occurs due to competitive binding for the operator-promoter in the prototypical mechanistic model between polymerase and transcription factor. It can be noted that k_T^{tl} matches with the model used in the RBS calculator [11] for the case that $w_{-4} \gg w_5 + w_4 R + \gamma_{tR}$ by neglecting degradation, assuming that only a small fraction of the ribosome binding sites are occupied and assuming that thermodynamic equilibrium occurs for the ribosome binding/unbinding.

The differential-algebraic equations are well defined as θ_{L1}, θ_{T1} are monotonically increasing functions of C_{2T}, C_{2L} for $0 \leq C_{2T} < g_T^T$, $0 \leq C_{2L} < g_L^T$ and so there is one non-negative solution for each function.

The model (6) can be used directly for numerical simulations, or can be further reduced to an ODE for either analytical or numerical analysis of the model. In order to find an ODE, we need to determine the inverse of θ_{L1}, θ_{T1} so that we have $C_{L2} = \theta_{L1}^{-1}(x_L^T)$ and similarly for $C_{T2} = \theta_{T1}^{-1}(x_T^T)$. This can be carried out numerically, if the parameters are known, or analytically using various (close) approximations (SI 2 and 3).

A simulation of (6) can be seen in Figure S1, which shows that the reduced DAE model is a (close) match to the full mechanistic model, assuming that the quasi-steady state approximation holds.

Nondimensionalisation and Standard Singular Perturbation Form

To determine under which conditions the quasi-steady state approximation holds, we need to apply singular perturbation theory [9]. The first step in this application is to nondimensionalise the original model, in order to determine which parameters are large or small.

We first transform the ODE, where the transformed equations with summed variables and conserved quantities are

$$\begin{aligned} \dot{m}_T^T &= \alpha_2(A_{1T} g_L^T - y_L^T) - \gamma_t m_T^T - (\gamma_{tR} - \gamma_t) C_{3T} \\ \dot{C}_{3T} &= w_4 R m_T^T - (w_4 R + w_{-4} + w_5 + \gamma_{tR}) C_{3T} \\ \dot{x}_T^T &= w_5 C_{3T} - \beta_t x_T^T - (\beta_{t2} - \beta_t) z_T^T - 2(\beta_{tg} - \beta_{t2}) C_{2T} \\ \dot{z}_T^T &= 2 w_6 \left(x_T^T - z_T^T\right)^2 - 2(w_{-6} + \beta_{t2}) \left(\frac{1}{2} z_T^T - C_{2T}\right) - 2\beta_{tg} C_{2T} \\ \dot{C}_{2T} &= w_8 \left(\frac{1}{2} z_T^T - C_{2T}\right) ((1 - A_{1T}) g_T^T + y_T - C_{2T}) - \left(w_{-8} + \beta_{tg}\right) C_{2T} \\ \dot{y}_T &= w_1 P C_{2T} - (w_1 P + w_{-1} + w_2) y_T \end{aligned} \tag{9}$$

where $y_T = A_{1T} g_T^T - C_{1T}$ is introduced as y_T is at a maximum steady state when C_{2T} is maximum, useful for scaling.

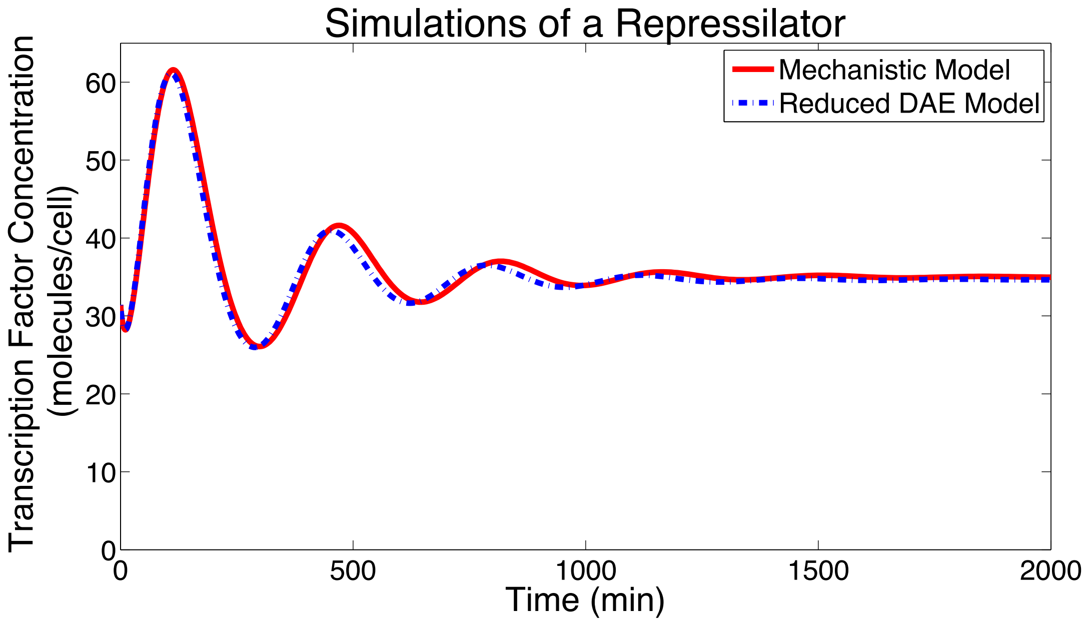

Figure S1: Simulations of the full mechanistic model (1) and reduced Differential Algebraic Equation (DAE) model (6) for a Repressilator network [5] with a stable equilibrium point. The reduced DAE model is a close approximation of the full mechanistic model when mRNA and protein degradation are much 'slower' than transcription, translation, operator binding and dimerisation. The parameters used for the simulation are $P = 1000, R = 1000, a_4 = 0.01, a_{-4} = 1, a_5 = 1, a_6 = 0.1, a_{-6} = 1, a_8 = 0.5, a_{-8} = 0.1, a_1 = 0.01, a_{-1} = 1, a_2 = 1, b_{L1} = 0.033, b_{L2} = 0.0167, b_{Lg} = 0.0167, \gamma_{LR} = 0.1, \gamma_{Lu} = 0.1$, with identical parameters for all three genes. The initial conditions were $m_T^T = m_Y^T = m_L^T = 0$, $X_{L10} = X_{T10} = 10, X_{Y10} = 20$, $g_L^T = g_Y^T = g_T^T = 1$, and other variables set at quasi-steady state (4).

The transformations to total transcription factor, total dimer and bound dimer allow the rate of change of each fast variable to (primarily) represent the dynamics of one reaction, e.g. transcription for $\dot{y}_T$ or dimerisation for $\dot{z}_T^T$. This representation allows simplified assumptions in terms of the individual reactions.

The dynamics of z_T^T and C_{2T} can be rewritten as

$$
\begin{aligned}
\dot{z}_T^T =& 2w_6 \left(x_T^T\right)^2 + 2(w_{-6} + \beta_{t2})C_{2T} \\
&- \left(2w_6 \left(2x_T^T - z_T^T\right) + w_{-6} + \beta_{t2}\right) z_T^T - 2\beta_{tg}C_{2T} \\
\dot{C}_{2T} =& \frac{1}{2}w_8((1 - A_{1T})g_T^T + y_T)z_T^T \\
&- \left(w_8 \left(\frac{1}{2}z_T^T - C_{2T}\right) + w_8(1 - A_{1T})g_T^T + w_8 y_T + w_{-8} + \beta_{tg}\right) C_{2T}
\end{aligned}
\tag{10}
$$

Notation: In the following section, $Y = Y_n \bar{Y}$ is used as notation for scaling, where Y is a variable, Y_n is a nondimensionalisation constant and $\bar{Y}$ is the non-dimensionalised variable.

We nondimensionalise all variables in reference to the maximum steady state over the range of inputs, which references the maximum gene expression level. Ribosome R and Polymerase P are typically referenced against their maximum value, but the initial value can also be used. It should be noted that m_T^T is minimum when y_L^T is maximum, and so we are not nondimensionalising using a particular equilibrium point. Using this reference, we have the nondimensionalisation parameters

$$
\begin{aligned}
M_{Tn}^T &= \frac{\alpha_2 A_{1L} g_L^T}{\gamma_{Tn}} \\
C_{3Tn} &= A_{2T} M_{Tn}^T \\
X_{Tn}^T &= \frac{w_5 C_{3Tn}}{\beta_{Tn}} \\
C_{2Tn} &= \theta_{T1}^{-1}(X_{Tn}^T) \\
Z_{Tn}^T &= \theta_{T2}(C_{2Tn}) = \theta_{T2}(\theta_{T1}^{-1}(X_{Tn}^T)) := \theta_{T3}(X_{Tn}^T) \\
Y_{1Tn} &= A_{1T} C_{2Tn} \\
\gamma_{Tn} &= \gamma_t(1 - A_{2T}) + \gamma_{tR} A_{2T} \\
\beta_{Tn} &= \beta_t + (\beta_{t2} - \beta_t)\eta_{Tm}^n + (\beta_{tg} - \beta_{t2})\eta_{Tg}^n \\
\eta_{Tm}^n &= \frac{Z_{Tn}^T}{X_{Tn}^T}, \quad \eta_{Tg}^n = \frac{2C_{2Tn}}{X_{Tn}^T}, \quad \eta_{To}^n = \frac{2C_{2Tn}}{Z_{Tn}^T} \\
t &= \frac{\tau}{\beta}
\end{aligned}
\tag{11}
$$

where β is the slow time scale. For simplicity of description, we also use

$$
\begin{aligned}
X_{Tn} &= X_{Tn}^T - Z_{Tn}^T \\
X_{T2n} &= Z_{Tn}^T - 2C_{2Tn} \\
g_{Tn} &= (1 - A_{1T})(g_T^T - C_{2tn})
\end{aligned}
\tag{12}
$$

where X_{Tn} is the monomeric transcription factor scaling constant, X_{T2n} is the dimeric transcription factor scaling constant and g_{Tn} is the free operator-promoter scaling constant.

When referencing x_T^T against the maximum, the scaling parameters are in implicit form, where θ_{T1}^{-1} and β are not stated explicitly. However, this approach holds as there is a unique, well defined, non-negative solution due to monotonically increasing θ_{T1}, θ_{T2} and $\beta_{Tn}X_{Tn}^T$ with respect to X_{Tn}^T. The parameters can be determined explicitly through either solving computationally, if the kinetic parameters are given, or by (close) approximation (see SI 2 and 3).

We can alternatively scale against a lower value for the total transcription factor in order to obtain a more accurate 'typical' maximum, such that

$$M_{Tn}^T = S_L \frac{\alpha_2 A_{1L} g_L^T}{\gamma_{Tn}} \tag{13}$$

where $0 \leq S_L \leq 1$ is a fraction dependent upon the typical operating range of C_{2L}, and thus dependent upon the entire gene regulatory network. Equivalently, we can scale against a reference regulation fraction $0 \leq F_{T,ref} < 1$, such that

$$X_{Tn}^T = X_{FT,ref} = \theta_{T1}(g_L^T F_{T,ref}) \tag{14}$$

where the other parameters are referenced against the new X_{Tn}^T, as per Equation (11).

Nondimensionalising the ODEs results in

$$\begin{aligned}
\frac{\beta}{\gamma_{Tn}}\dot{\bar{m}}_T^T =&(1 - A_{3L}\bar{y}_L) - \frac{\gamma_t}{\gamma_{Tn}}\bar{m}_T^T - \frac{\gamma_{Tn} - \gamma_t}{\gamma_{Tn}}\bar{C}_{3T} \\
\frac{\beta}{\beta_{Tn}}\dot{\bar{x}}_T^T =&\bar{C}_{3T} - \frac{\beta_t}{\beta_{Tn}}\bar{x}_T^T - \frac{\beta_{t2} - \beta_t}{\beta_{Tn}}\eta_{Tm}^n \bar{z}_T^T - \frac{\beta_{tg} - \beta_{t2}}{\beta_{Tn}}\eta_{Tg}^n \bar{C}_{2T} \\
\varepsilon_{w1}\dot{\bar{C}}_{3T} =&\bar{m}_T^T - \bar{C}_{3T} \\
\varepsilon_{w2}\dot{\bar{y}}_T =&\bar{C}_{2T} - \bar{y}_T \\
\varepsilon_{w3}\dot{\bar{z}}_T =&A_{4T}A_{5T}\left(\bar{x}_T^T\right)^2 + A_{4T}(1 - A_{5T})\bar{C}_{2T} \\
&- \left(A_{6T}\bar{x}_T - \frac{1}{2}A_{7T}\bar{z}_T^T + 1 - A_{6T} + A_{7T}\right)\bar{z}_T^T - \varepsilon_{w5}\bar{C}_{2T} \\
\varepsilon_{w4}\dot{\bar{C}}_{2T} =&A_{8T}(A_{9T} + (1 - A_{9T})\bar{y}_T)\bar{z}_T^T \\
&- \left(A_{10T}\bar{z}_T^T - \frac{1}{2}A_{11T}\bar{C}_{2T} + A_{12T}\bar{y}_T + 1 - A_{10T} + A_{11T} - A_{12T}\right)\bar{C}_{2T}
\end{aligned} \tag{15}$$

with parameters

$$\begin{aligned}
\beta_{C3T} &= w_4 R_n + w_{-4} + w_5 + \gamma_{tR} \\
\beta_{YT} &= w_1 P_n + w_{-1} + w_2 \\
\beta_{C2T} &= \frac{1}{2}w_8(Z_{Tn}^T - 2C_{2tn}) + w_8(1 - A_{1T})(g_T^T - C_{2tn}) + w_{-8} + \beta_{tg} \\
&= w_8 X_{T2n} + w_8 g_{Tn} + w_{-8} + \beta_{tg} \\
\beta_{ZT} &= 4w_6(X_{Tn}^T - Z_{Tn}^T) + w_{-6} + \beta_{t2} \\
&= 4w_6 X_{Tn} + w_{-6} + \beta_{t2} \\
\varepsilon_{w1} &= \frac{\beta}{\beta_{C3T}}, \quad \varepsilon_{w2} = \frac{\beta}{\beta_{yT}}, \quad \varepsilon_{w3} = \frac{\beta}{\beta_{C2T}}, \quad \varepsilon_{w4} = \frac{\beta}{\beta_{ZT}}, \quad \varepsilon_{w5} = \frac{\beta_{tg}\eta_{To}}{\beta_{ZT}}
\end{aligned} \tag{16}$$

$$
\begin{aligned}
A_{3L} &= \frac{C_{2Ln}}{g_L^T}, \quad A_{4T} = 1 + \frac{2w_6 X_{Tn}}{\beta_{ZT}}, \quad A_{5T} = \frac{2w_6 \left(X_{Tn}^T\right)^2}{2w_6 \left(X_{Tn}^T\right)^2 + 2(w_{-6} + \beta_{t2})C_{2Tn}} \\
A_{6T} &= \frac{4w_6 X_{Tn}^T}{\beta_{ZT}}, \quad A_{7T} = \frac{4w_6 Z_{Tn}^T}{\beta_{ZT}} \\
A_{8T} &= 1 + \frac{w_8 C_{2Tn}}{\beta_{C2T}}, \quad A_{9T} = \frac{w_8(1 - A_{1T})g_T^T}{w_8(1 - A_{1T})g_T^T + A_{1T}C_{2Tn}} \\
A_{10T} &= \frac{\frac{1}{2}w_8 Z_{Tn}^T}{\beta_{C2T}}, \quad A_{11T} = \frac{2w_8 C_{2Tn}}{\beta_{C2T}}, \quad A_{12T} = \frac{w_8 Y_{Tn}}{\beta_{C2T}}
\end{aligned}
$$

noting that we cancel terms as the nondimensionalisation is based on the maximum steady state for each variable. We estimate the separate time-scales $\beta_{C3T}, \beta_{ZT}, \beta_{YT}, \beta_{C2T}$ of the individual fast variables from the diagonal of the Jacobian matrix of the fast variables about the candidate slow manifold.

To place the system in standard form, we need to estimate a lower bound for the fast time-scale, which we achieve based on local analysis about the candidate slow manifold. This lower bound is required to be slower than the individual fast variables, taking any coupling into account between the variables. C_{3T} is uncoupled from other variables on the fast time scale, while y_T,C_{2T},z_T^T are coupled. From experimental observations, we can typically assume that $\beta_{C2T} \ll \beta_{C1T}$ [7], and so β_{C1T} is effectively decoupled. By using estimates of the minimum time scale based on the eigenvalue analysis, which hold locally about the quasi-steady state, we can estimate the lowest fast time scale to be

$$
\begin{aligned}
\beta_{coup} &= \frac{\beta_{ZT} + \beta_{C2T}}{2} - \sqrt{\left(\frac{\beta_{ZT} + \beta_{C2T}}{2}\right)^2 - \left[\beta_{C2T}\beta_{ZT} - w_8 g_{Ln}(w_{-6} + \beta_{t2})\right]} \\
\varepsilon_{w6} &= \frac{\beta}{\beta_{coup}}
\end{aligned}
\tag{17}
$$

setting ε_{w6}, as we need $\beta \ll \beta_{coup}$ for time-scale separation to occur. These coupling conditions ensure that as well as both C_{2T} and z_T^T being fast variables, all possible local transformations of the two variables are also fast. If required, we can generalise the assumptions for the case that y_T,C_{2T},z_T^T are on the same time scale, which we can once again achieve using local analysis.

We estimate the slow time-scale using

$$
\beta = \max\left(\beta_{Tn}, \gamma_{Tn}\right) \tag{18}
$$

The 'slow' time scale may differ for a network compared to a 'module' analysed here, with the network typically slower. However, the network may be faster due to feedback e.g. auto regulation [1].

In order to obtain useful estimates of fast and slow time-scales, care is required in selecting total transcription factor scaling X_{Tn}^T using (11) or (13), which should be relevant to the behaviour of the entire network. A choice of X_{Tn}^T well above the concentration range relevant for regulation of expression could result in an overestimate of the speed of dimerisation or operator binding. For example, for a large X_{Tn}^T where almost all operators are occupied (resulting in $C_{2Ln} = 0.999g_L^T$) and where there is an excess of free dimeric transcription factor X_{2Tn}, then the estimated speed of operator binding may be orders of magnitude too high compared to a lower but still typical level of operator binding ($C_{2Ln} = 0.8g_L^T \sim g_L^T$). Similarly, if X_{Tn}^T is too low, then there may be an unnecessary underestimate. For example, if dimerisation is (conservatively) required to be fast for all X_{Tn}^T, then the speed of dimerisation may be estimated

with a lower bound $\beta_{ZT} \geq w_{-6} + \beta_{t2}$ based on very low transcription factor concentrations. However, this lower bound is often likely to significantly underestimate the dimerisation time scale at concentrations relevant to regulation, which is typically when $C_{2Ln} \sim g_L^T$, depending upon the allowed error.

We can place (15) in standard singular perturbation form [9]

$$\begin{aligned} \dot{x}_1 &= f_1(t, x_1, x_2, \varepsilon) \\ \varepsilon \dot{x}_2 &= f_2(t, x_1, x_2, \varepsilon) \end{aligned} \tag{19}$$

by setting $x_1 = (\bar{m}_T^T, \bar{x}_T^T)$, $x_2 = (\bar{C}_{3T}, \bar{y}_T, \bar{z}_T^T, \bar{C}_{2T})$, and $\varepsilon = \max(\varepsilon_{w1}, \ldots, \varepsilon_{w6})$. By symmetry, the same methodology applies for reactions involving $X_L, X_{L2}, g_L X_{L2}, g_L P$, using equivalent assumptions, which can be used to derive the gene expression of x_T^T.

From the standard form (19), we can see that ε is small and thus that Quasi-Steady State holds under the assumptions

$$\begin{aligned} \beta_{Tn}, \gamma_{Tn} \ll &(w_4 R_n + w_{-4} + w_5 + \gamma_{tR}), (w_1 P_n + w_{-1} + w_2), \\ &(w_8 X_{T2n} + w_8 g_{Tn} + w_{-8} + \beta_{tg}), (4 w_6 X_{Tn} + w_{-6} + \beta_{t2}) \end{aligned} \tag{20}$$

as well as

$$\beta_{tg} \eta_{To} \ll 4 w_6 X_{Tn} + w_{-6} + \beta_{t2} \tag{21}$$

and $\beta_{C2T} \ll \beta_{C1T}$ together with $\beta_{Tn}, \gamma_{Tn} \ll \beta_{coup}$, where the last of which can be relaxed to

$$\beta_{Tn}, \gamma_{Tn} \ll \frac{\beta_{C2T} \beta_{ZT} - w_8 g_{Tn} (w_{-6} + \beta_{t2})}{\beta_{C2T} + \beta_{ZT}} \tag{22}$$

when (20) holds.

The conditions in (20) require that the sum of the forward and reverse rates for transcription, translation, operator binding and dimerisation are faster than mRNA and protein degradation. For dimerisation and operator binding, this holds if either the forward or the reverse rates are sufficiently fast compared to degradation. For transcription and translation, this condition holds if ribosome/polymerase binding, unbinding or initiation is sufficiently fast.

Equation (22) holds if (20) holds unless both the reverse rate of operator binding and the forward rate of dimerisation are relatively small, when the operator is not being fully occupied. For the case that (22) does not hold, there would be a near zero free dimer concentration at quasi-steady state, which would limit bound dimer ($X_{T2} g_T$) and monomer (X_T) reaching (quasi) equilibrium on a fast time scale. This case is typically only possible at concentrations too low to be of interest for modelling of regulation.

The derived conditions generalise the existing model reduction methodology that uses total transcription factor indirectly [8, 6], where the generalisation is required to match with known experimental data. In [7], the isolated forward rate of chromosomal lacI-operator binding has an associated time-scale of $\approx \frac{1}{2}$ min, while the reverse rate has an associated time-scale of $\approx$ 10 min, noting that lacI is a dimer of dimers and that $X_{Tn}^T \approx$ 20 molecules/cell [4]. As $\gamma_T \approx$ 5 min and β_T has a time scale between 5 min and hours/days [1], then experimental results indicate that in a typical case for lacI, only the forward rate is fast enough for time-scale separation to hold generally. As the assumptions used here require either a fast forward or reverse rate, the current reduced model assumptions are consistent with experimental data for lacI [4, 7].

The time-scale separation conditions may be conservative for the asymptotic cases $X_{Tn} \ll X_{Tn}^T$ or $X_{T2n} \ll X_{Tn}^T$ or $g_{Tn} \ll g_T^T$, as for these cases the local analysis may not contain a useful

estimate of the time-scales. For example, if $X_{Tn} \ll X_{Tn}^T$ holds and $\beta_{ZT} \gg \beta_{Tn}, \gamma_{Tn}$ does not hold, but the latter holds for a hypothetical $X_{Tn} \sim X_{Tn}^T$, then X_{Tn} typically converges to near zero sufficiently quickly such that the error in degradation rate of x_T^T and quasi-steady state of C_{2T} are small. As C_{2T} is a proxy variable for regulation, the quasi-steady state is a useful estimate of regulation for this case.

Equivalent time-varying equations may be obtained if R and P are time-varying parameters, such that

$$\begin{aligned} \varepsilon_{w1}\dot{\bar{C}}_{3T} &= \bar{R}\bar{m}_T^T - (A_{2T}\bar{R} + (1 - A_{2T}))\bar{C}_{3T} - \varepsilon_{w5}\bar{C}_{3T} \\ \varepsilon_{w2}\dot{\bar{y}}_T &= \bar{P}\bar{C}_{2T} - (A_{1T}\bar{P} + (1 - A_{1T}))\bar{y}_T \end{aligned} \tag{23}$$

In this case, we also require that P and R are slowly time-varying. We require $\frac{d\bar{P}}{d\tau_f} \ll 1$ and $\frac{d\bar{R}}{d\tau_f} \ll 1$, where τ_f is the fast time scale. This is equivalent to the unscaled conditions

$$\varepsilon\dot{P} \ll \beta P_n, \quad \varepsilon\dot{R} \ll \beta R_n \tag{24}$$

These conditions on parameters R and P in (24) hold locally about the slow manifold, and hold on the entire domain when there is a sufficiently large number of polymerase/ribosomes such that binding has little effect on the free polymerase/ribosomes concentration.

Application of Tikhonov's Theorem

To show that the quasi-steady state approximation holds, we need to show that the conditions for a version of Tikhonov's Theorem are met on the domain of interest [9], which for this case is the set of non-negative values of each concentration below the maximum of each variable. As stated earlier, the non-dimensionalised quasi-steady state has a unique solution. The well defined nature of $\bar{C}_{2T}$ in terms of $\bar{x}_T^T$ on the domain of interest implies an isolated root to the quasi-steady state solution. The functions f_1 and f_2 in (19) are polynomial in terms of (x_1, x_2, ε) and so are sufficiently smooth. Treating R and P as time-varying parameters, function f_2 is sufficiently smooth with respect to x_T^T if R and P are continuously differentiable. The inverse function theorem implies that the function $\bar{C}_{2T} = \theta_{T1}^{-1}(X_{Tn}^T\bar{x}_T^T)/C_{2Tn}$ is sufficiently smooth on the domain for the reduced problem to have a well defined solution. Finally, for the fast dynamics it can be shown that the isolated root is (locally) exponentially stable by using local eigenvalue analysis. We set

$$f_2(t, x_1, x_2, \varepsilon) = \varepsilon \begin{pmatrix} \dot{\bar{C}}_{3T} \\ \dot{\bar{C}}_{1T} \\ \dot{\bar{z}}_T \\ \dot{\bar{C}}_{2T} \end{pmatrix} \tag{25}$$

If R and P are time-varying, then they are assumed slowly time-varying and we can set $R = R(0)$ and $P = P(0)$ for the boundary value problem. The linearisation of the scaled boundary value problem is

$$\frac{\partial f_2}{\partial x_2}(x_1, x_2, 0) = \begin{pmatrix} \frac{\varepsilon}{\varepsilon_1} & 0 & 0 & 0 \\ 0 & \frac{\varepsilon}{\varepsilon_2} & 0 & 0 \\ 0 & 0 & \frac{\varepsilon}{\varepsilon_3} & 0 \\ 0 & 0 & 0 & \frac{\varepsilon}{\varepsilon_4} \end{pmatrix} \begin{pmatrix} -1 & 0 & 0 & 0 \\ 0 & -1 & 0 & 1 \\ 0 & 0 & J_{33} & J_{34} \\ 0 & J_{42} & J_{43} & J_{44} \end{pmatrix} \tag{26}$$

where

$$
\begin{aligned}
J_{33} =& -\left(A_{6T}\bar{x}_T^T - A_{7T}\bar{z}_T^T + 1 - A_{6T} + A_{7T}\right)\\
J_{34} =& A_{4T}(1 - A_{5T})\\
J_{42} =& A_{8T}(1 - A_{9T})\bar{z}_T^T - A_{2T}\bar{C}_{2T}\\
J_{43} =& A_{8T}(A_{9T} + (1 - A_{9T})\bar{y}_T) - A_{10T}\bar{C}_{2T}\\
J_{44} =& -\left(A_{10T}\bar{z}_T - A_{11T}\bar{C}_{2T} + A_{12T}\bar{y}_T + 1 - A_{10T} + A_{11T} - A_{12T}\right)
\end{aligned}
\tag{27}
$$

The matrix is Hurwitz for the entire domain of interest, and so the isolated root is (locally) exponentially stable. Thus the conditions for Tikhonov's Theorem are met internally on the domain of interest [9].

For the boundary cases, the isolated root is only on the boundary of the domain for $x_T^T = 0$ or $m_T^T = 0$, on the assumption that the kinetic rates are positive constants. We note that $x_T^T = 0$ implies $x_T^T = z_T^T = C_{2T} = y_T = 0$ and so the quasi-steady state approximation holds trivially for $C_{2T}, x_T^T, z_T^T, y_T$ on the boundary of the domain, as well as holding in the limit due to continuity. This logic similarly holds for $m_T^T = 0$. Thus for sufficiently small ε in (19) then the quasi-steady state approximation holds on the domain.

2 Simplified Expression Model

In this section, we simplify the model of expression for gene regulation from an implicit to an explicit form, which together with simplified degradation (SI 3), enables us to represent the reduced model as an ODE rather than a DAE (differential-algebraic equation). To derive an ODE, we need to determine the expression in terms of the total TF (Transcription Factor), which is an inverse function problem. To solve this, we partition the function into multiple cases and then use Padé approximations to approximate the inverse function. The two cases used for the partition are the monomer dominant and multimer dominant regulation, which indicate the predominant form of the regulating transcription factor at concentrations relevant for regulation. We use two methods of perturbation theory and interpolation to determine the Padé approximations and to determine the partitioning of the cases. Using interpolation is conceptually related to previous interpolation methods for Michaelis-Menten kinetic derivations [3].

To determine expression explicitly, we need to invert the function $\theta_{L1}(C_{2L})$, where

$$
x_L^T = \theta_{L1}(C_{2L}) := \frac{\sqrt{C_{2L}}}{\sqrt{B_{L2}B_{Lg}(g_L^T - C_{2L})}} + \frac{2C_{2L}}{B_{Lg}(g_L^T - C_{2L})} + 2C_{2L} \tag{28}
$$

2.1 Perturbation Theory

We initially approximate the inverse for the multimer dominant case, where

$$
x_L^T \approx z_L^T = \frac{2C_{2L}}{B_{Lg}(g_L^T - C_{2L})} + 2C_{2L} \tag{29}
$$

and the monomer dominant case, where

$$
x_L^T \approx X_L = \frac{\sqrt{C_{2L}}}{\sqrt{B_{L2}B_{Lg}(g_L^T - C_{2L})}} \tag{30}
$$

before using perturbation theory to both determine when the two cases are valid, and to find more accurate approximations.

We provide the approximation in terms of the multimerisation efficiency $\eta_{Lm} := \frac{z_L^T}{x_L^T}$, which is the fraction of a protein in its full multimer form. There are alternative approaches to represent the approximation than using the multimerisation efficiency. However, we use the approach presented here as it is relatively simple and allows the refined approximations to have a simple biological interpretation.

2.1.1 Multimer Dominant Regulation

We first look at the multimer dominant case. Using $z_L^T = \eta_{Lm} x_L^T$, we have

$$\begin{aligned} C_{2L} &= \frac{g_L^T}{2} + \frac{\eta_{Lm} x_L^T}{4} + \frac{1}{2B_{Lg}} - \sqrt{\left(\frac{g_L^T}{2} + \frac{\eta_{Lm} x_L^T}{4} + \frac{1}{2B_{Lg}}\right)^2 - \frac{g_L^T \eta_{Lm} x_L^T}{2}} \\ &= \frac{g_L^T}{2} + \frac{\eta_{Lm} x_L^T}{4} + \frac{1}{2B_{Lg}} - \sqrt{\left(\frac{\eta_{Lm} x_L^T}{4} + \frac{1}{2B_{Lg}} - \frac{g_L^T}{2}\right)^2 + \frac{g_L^T}{B_{Lg}}} \end{aligned} \tag{31}$$

and so

$$\begin{aligned} F_L &= \frac{g_L^T - C_{2L}}{g_L^T} \\ &= \sqrt{\left(\frac{\eta_{Lm} x_L^T}{4 g_L^T} + \frac{1}{2B_{Lg} g_L^T} - \frac{1}{2}\right)^2 + \frac{1}{B_{Lg} g_L^T}} - \left(\frac{\eta_{Lm} x_L^T}{4 g_L^T} + \frac{1}{2B_{Lg} g_L^T} - \frac{1}{2}\right) \end{aligned} \tag{32}$$

We set the initial approximation

$$F_{L0}(x_L^T) := F_L|_{\eta_{Lm} = \eta_{Lm0}} \tag{33}$$

where $\eta_{Lm0} = 1$.

More formally, we can write out the non-dimensionalised algebraic equation in (28) using the scaling approximation $C_{2Ln} = g_L^T$ (noting $C_{2L} = C_{2Ln}\bar{C}_{2L}$) to obtain the inverse function perturbation problem

$$\begin{aligned} & A_1 \bar{C}_{2L} + (1 - A_1)\frac{\bar{C}_{2L}}{1 - \bar{C}_{2L}} + \varepsilon_L \sqrt{\frac{\bar{C}_{2L}}{1 - \bar{C}_{2L}}} = A_2 \\ & A_1 = \frac{B_{Lg} g_L^T}{1 + B_{Lg} g_L^T} \\ & A_2 = \frac{B_{Lg} x_L^T}{2(1 + B_{Lg} g_L^T)} \\ & \varepsilon_L = \frac{\sqrt{B_{Lg}}}{2(1 + B_{Lg} g_L^T)\sqrt{B_{L2}}} \end{aligned} \tag{34}$$

There is a boundary layer near $\bar{C}_{2L} = 1$ if $1 - A_1 = O(\varepsilon)$, but we are only interested in the outer solution.

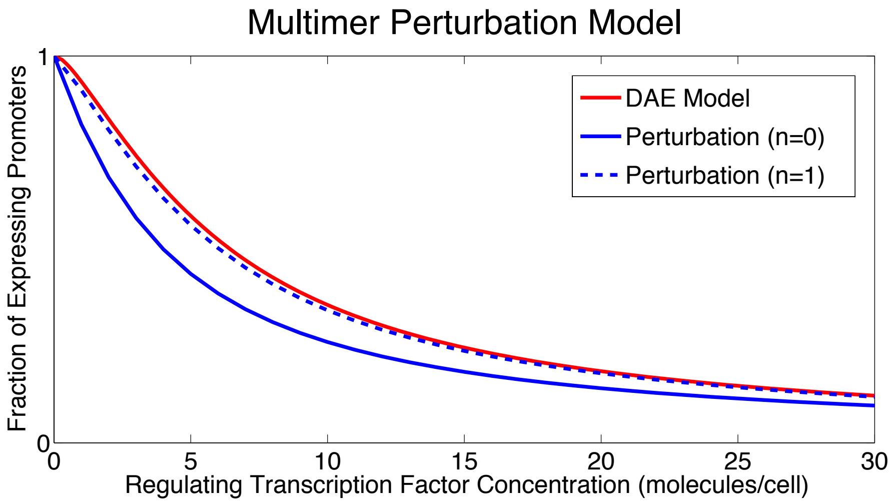

Figure S2: Comparison of Regulation function F_L from the DAE (6) and perturbation approach (33) for the multimer dominant case. The parameters are $g_L^T = 1$, $\frac{1}{B_{L2}} = 4$ and $\frac{1}{B_{Lg}} = 1$ (molecules/cell).

We transform this perturbation problem to be in terms of the multimerisation efficiency, such that

$$\eta_{Lm} A_2 + \varepsilon_L \sqrt{\bar{F}_{L0} \left(\eta_{Lm} A_2\right)^{-1} - 1} = A_2 \tag{35}$$

where $\bar{F}_{L0}(\eta_{Lm} A_2) = F_{L0}(\eta_{Lm} x_L^T)$.

Padé Approximation - Multimer Case

We next use a Padé approximation to estimate the multimerisation efficiency, as the Padé approximation is a close approximation for a large range of ε_L. In comparison, a 1st order Taylor series is much slower to converge and predicts negative solutions for small A_2 (i.e. small x_L^T), which is not biologically feasible. For the zeroth order Padé approximation, we have $\eta_{Lm0} = 1$, which is the 'multimeric transcription factor only' case. Using a first order Padé approximation $\eta_{Lm1} = \frac{a_0(A_2)}{1+\varepsilon a_1(A_2)}$ and matching to the Taylor series, we have

$$\eta_{Lm1} = \frac{A_2}{A_2 + \varepsilon_L \sqrt{\bar{F}_{L0} \left(A_2\right)^{-1} - 1}} \tag{36}$$

Using the original scaling and variables, we have

$$\eta_{Lm1} = \frac{x_L^T}{x_L^T + \frac{1}{\sqrt{B_{L2} B_{Lg}}} \sqrt{F_{L0} \left(x_L^T\right)^{-1} - 1}} \tag{37}$$

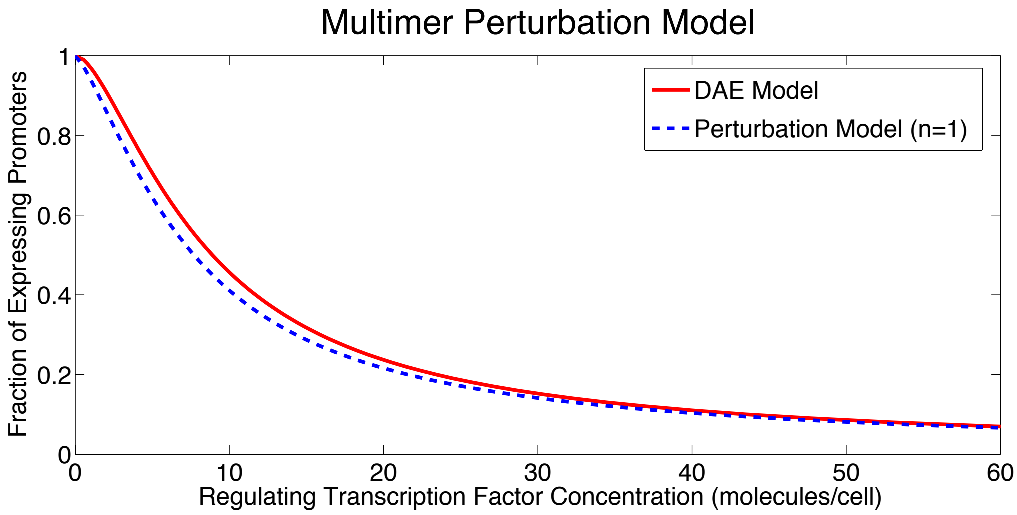

Figure S3: Comparison of Regulation function F_L from the DAE (6) and perturbation model (33) for the multimer dominant case. Parameters are $g_L^T = 1$, $\frac{1}{B_{Lg}} = 1$, $\frac{1}{B_{L2}} = 16$ (molecules/cell) and $\varepsilon_L = 1$. The parameters chosen give the boundary case $\varepsilon_L = 1$, but the first order Padé approximation only has a small error from the full mechanistic model.

2.1.2 Monomer Dominant Regulation

We next look at the monomer dominant case. Using $x_L = (1 - \eta_{Lm})x_L^T$, we have

$$(1 - \eta_{Lm})x_L^T = \frac{\sqrt{C_{2L}}}{\sqrt{B_{L2}B_{Lg}(g_L^T - C_{2L})}} \tag{38}$$

and so

$$F_L((1 - \eta_{Lm})x_L^T) = \frac{g_L^T - C_{2L}}{g_L^T} = \frac{1}{1 + B_{Lg}B_{L2}(1 - \eta_{Lm})^2 {x_L^T}^2} \tag{39}$$

We again set

$$F_{L0}(x_L^T) := F_L|_{\eta_{Lm} = \eta_{Lm0}} \tag{40}$$

but use $\eta_{Lm0} = 0$.

We find the non-dimensionalised algebraic equation in (6) using the approximation $C_{2Ln} = g_L^T$ to obtain the perturbation problem

$$\begin{aligned}
&\sqrt{\frac{\bar{C}_{2L}}{1 - \bar{C}_{2L}}} + \varepsilon_{Ls}\left(A_1\bar{C}_{2L} + (1 - A_1)\frac{\bar{C}_{2L}}{1 - \bar{C}_{2L}}\right) = A_2 \\
&A_1 = \frac{B_{Lg}g_L^T}{1 + B_{Lg}g_L^T} \\
&A_2 = x_L^T\sqrt{B_{L2}B_{Lg}} \\
&\varepsilon_{Ls} = 2(1 + B_{Lg}g_L^T)\sqrt{\frac{B_{L2}}{B_{Lg}}} = \frac{1}{\varepsilon_L}
\end{aligned} \tag{41}$$

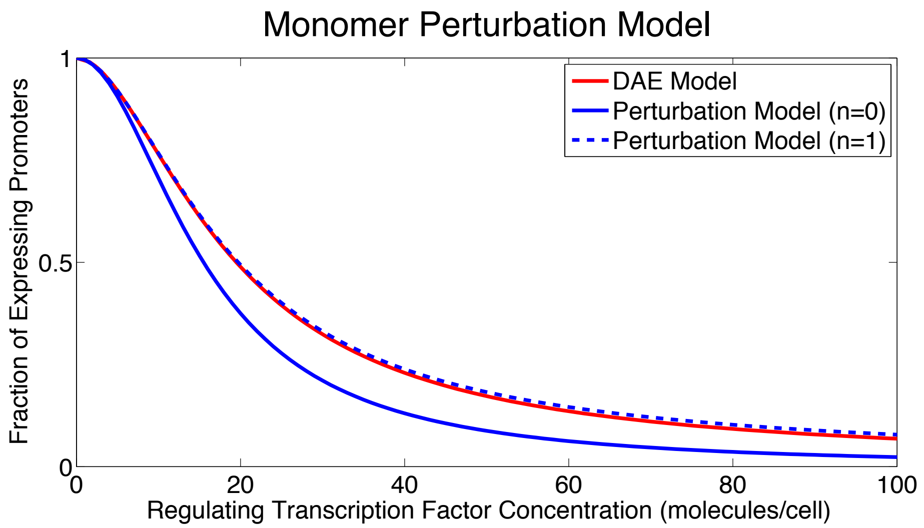

Figure S4: Comparison of Regulation function F_L from the DAE (6) and perturbation model (39) for the monomer dominant case. Parameters are $g_L^T = 1, \frac{1}{B_{Lg}} = 1.5, \frac{1}{B_{L2}} = 150$ (molecules/cell) and $\varepsilon_L \approx 3.1$.

For leading order behaviour of order 1 as $\bar{C}_{2L} \to 0$, we square the perturbation problem, such that

$$\begin{aligned}&\frac{\bar{C}_{2L}}{1-\bar{C}_{2L}} + 2\varepsilon_{Ls}\sqrt{\frac{\bar{C}_{2L}}{1-\bar{C}_{2L}}}\left(A_1\bar{C}_{2L} + (1-A_1)\frac{\bar{C}_{2L}}{1-\bar{C}_{2L}}\right)\\&+\varepsilon_{Ls}^2\left(A_1\bar{C}_{2L} + (1-A_1)\frac{\bar{C}_{2L}}{1-\bar{C}_{2L}}\right)^2 = A_2^2\end{aligned} \tag{42}$$

Using $X_L^2 = h_L {x_L^T}^2$, we have

$$\begin{aligned}&h_L A_2^2 + 2\varepsilon_{Ls}\sqrt{h_L}A_2\left(A_1(1-\bar{F}) + (1-A_1)(\bar{F}^{-1}-1)\right)\\&+\varepsilon_{Ls}^2\left(A_1(1-\bar{F}) + (1-A_1)(\bar{F}^{-1}-1)\right)^2 = A_2^2\end{aligned} \tag{43}$$

Solving this, we have $h_{L0} = 1$ as expected, as well as

$$h_{L1} = \frac{A_2}{A_2 + 2\varepsilon_{Ls}\left(A_1(1-\bar{F}_0) + (1-A_1)(\bar{F}_0^{-1}-1)\right)} \tag{44}$$

With original scaling and parameters, this becomes

$$h_{L1} = \frac{x_L^T}{x_L^T + 4\left(g_L^T(1-F_0) + \frac{1}{B_{Lg}}(F_0^{-1}-1)\right)} \tag{45}$$

and we set $h_{L1} = (1-\eta_{Lm1})^2$.

2.1.3 Alternative to Monomer Dominant Case

We can describe the expression term in Equation 2 (main paper) in an alternative representation by using the overlapping cases of predominantly multimer and predominantly free monomer/dimer, such that

$$
\begin{aligned}
F_L &= \begin{cases} \sqrt{W_L^2 + \frac{1}{B_{Lg} g_L^T}} - W_L, & \varepsilon_L \leq 1 \\ \frac{1}{1+\frac{B_{Lg}}{16 B_{L2}}\left(\sqrt{1+8B_{L2} h_L x_L^T}-1\right)^2}, & \varepsilon_L > 1 \end{cases} \\
W_L &= \frac{\eta_{Lm} x_L^T}{4 g_L^T} + \frac{1}{2 B_{Lg} g_L^T} - \frac{1}{2}, \quad \varepsilon_L = \frac{\sqrt{B_{Lg}}}{2(1 + B_{Lg} g_L^T)\sqrt{B_{L2}}}
\end{aligned}
\tag{46}
$$

where the free transcription factor fraction h_L replaces the multimerisation efficiency η_{Lm}.

However, here we use the original regulation term (Equation 3 in the paper), at least for lower order approxations, for simplicity and for the ability to more easily relate the proposed regulation term to traditional models. It can be noted that modelling the total free monomer/dimer concentration is of use to determining reduced models for tetrameric TF.

For the free monomer/dimer case, we use the perturbation problem

$$
\begin{aligned}
A_1 \sqrt{\frac{\bar{C}_{2L}}{1-\bar{C}_{2L}}} + (1 - A_1)\frac{\bar{C}_{2L}}{1-\bar{C}_{2L}} + \varepsilon_{Lu} \bar{C}_{2L} &= A_2 \\
A_1 &= \frac{1}{1 + 2\sqrt{\frac{B_{L2}}{B_{Lg}}}} \\
A_2 &= \frac{B_{Lg}}{2} x_L^T \frac{1}{1 + 2\sqrt{\frac{B_{L2}}{B_{Lg}}}} \\
\varepsilon_{Lu} &= 2 g_L^T \frac{\sqrt{B_{L2} B_{Lg}}}{1 + 2\sqrt{\frac{B_{L2}}{B_{Lg}}}}
\end{aligned}
\tag{47}
$$

noting that $\varepsilon_{Lu} \leq \varepsilon_{Ls} = \frac{1}{\varepsilon_L}$, and so (47) covers the case of monomer dominant regulation. Using overlapping perturbation problems allows a non-unique partitioning, and so we can also replace ε_L with $1/\varepsilon_{Lu}$ if required.

Solving, we have

$$
h_{L0} = 1 \tag{48}
$$

Using the same methodology as the multimer dominant case, the first order (unscaled) Padé approximation is

$$
h_{L1} = \frac{x_L^T}{x_L^T + 2 g_L^T (1 - F_{L0})} \tag{49}
$$

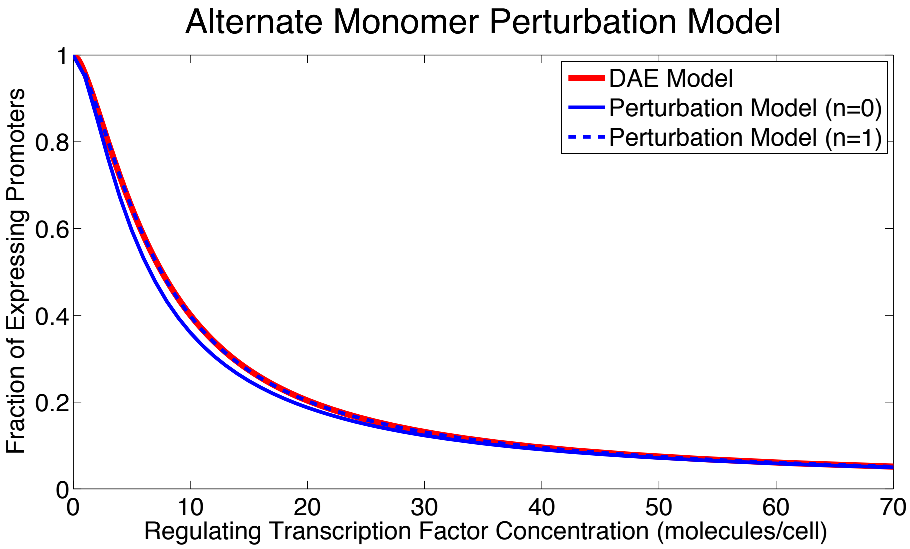

Figure S5: Comparison of Regulation function F_L from the DAE (6) and perturbation model (46) for the free transcription factor case. Parameters are $g_L^T = 1, \frac{1}{B_{Lg}} = 1.4, \frac{1}{B_{L2}} = 10.2$ (molecules/cell) and $\varepsilon_L \approx 0.78$.

2.2 Interpolation

We also use interpolation to determine the Padé approximation and to determine the required case, such that

$$\begin{aligned} k_T^{tx} &= V_T^{tx} F_L \\ F_L &= \begin{cases} \sqrt{W_L^2 + \frac{1}{B_{Lg} g_L^T}} - W_L, & \eta_{Lm0} \geq \frac{1}{2} \\ \frac{1}{1+B_{Lg}B_{L2}h_L x_L^{T\,2}}, & \eta_{Lm0} < \frac{1}{2} \end{cases} \\ W_L &= \frac{\eta_{Lm} x_L^T}{4 g_L^T} + \frac{1}{2 B_{Lg} g_L^T} - \frac{1}{2} \end{aligned} \tag{50}$$

This test determines the predominant form of transcription factor when half of the operators are occupied.

To approximate multimerisation efficiency for the multimer dominant case, we use the point $C_{2g} = \frac{1}{2} g$ for a one point interpolation, such that

$$\eta_{Lm0} = \frac{\sqrt{\frac{B_2}{B_{Lg}}}(2 + g_L^T B_{Lg})}{1 + \sqrt{\frac{B_2}{B_{Lg}}}(2 + g_L^T B_{Lg})} \tag{51}$$

Similarly, for the first order rational case, we interpolate the points $C_{2g} = 0$, $C_{2g} = \frac{1}{2} g$, and $C_{2g} \to g$, which gives

$$\begin{aligned} \eta_{Lm1} &= \frac{x_L^T}{x_L^T + A_{Lm}} \\ A_{Lm} &= \frac{1}{\eta_{Lm0}\sqrt{B_{L2} B_{Lg}}} = \frac{1 + \sqrt{\frac{B_2}{B_{Lg}}}(2 + g_L^T B_{Lg})}{B_{L2}(2 + g_L^T B_{Lg})} \end{aligned} \tag{52}$$

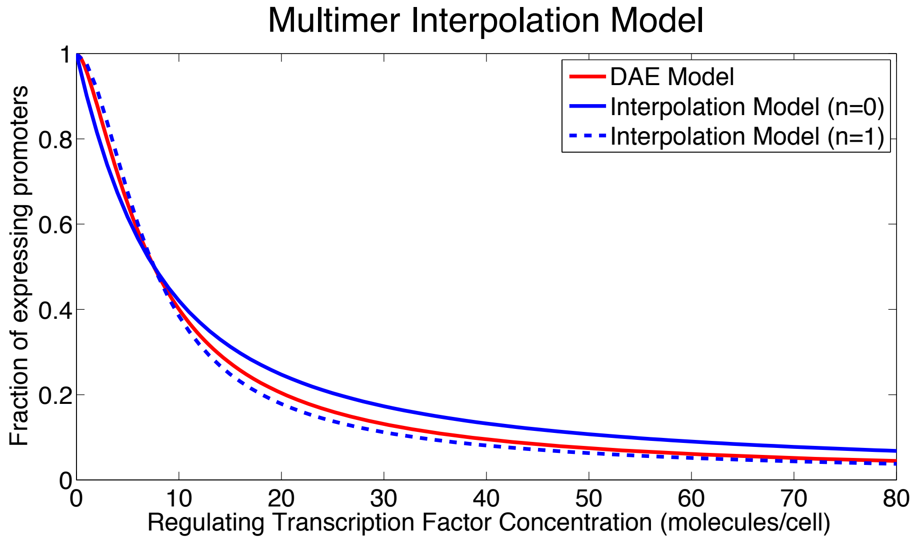

Figure S6: Comparison of DAE model (6) and multimer interpolation model (50), using parameters $g_L^T = 1$, $\frac{1}{B_{Lg}} = 1.4$, $\frac{1}{B_{L2}} = 10.2$ (molecules/cell), where $\eta_{Lm0} = 0.50$.

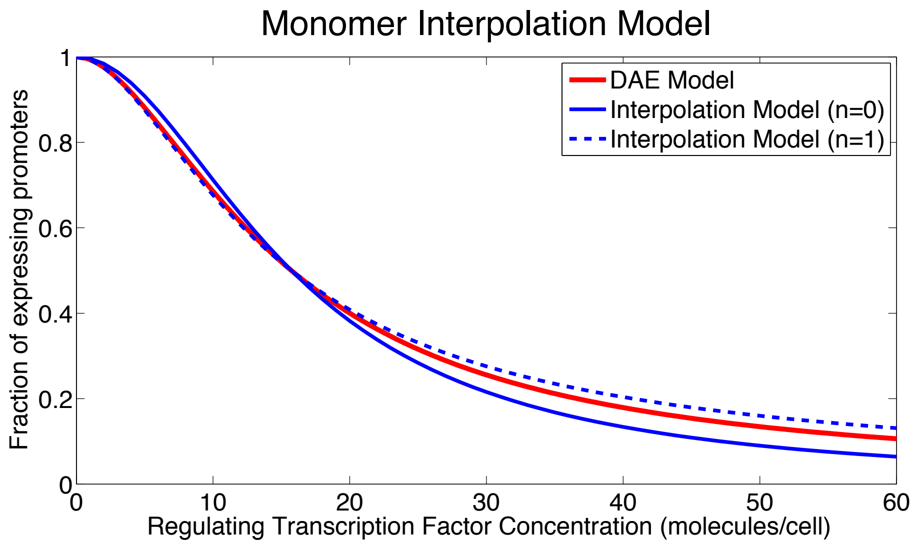

Figure S7: Comparison of DAE model (6) and monomer interpolation model (50), using parameters $g_L^T = 1$, $\frac{1}{B_{Lg}} = 1.4$, $\frac{1}{B_{L2}} = 101$ (molecules/cell), where $\eta_{Lm0} = 0.24$.

For the monomer dominant case, we use an identical zeroth order approximation to the multimer case, where

$$h_{L0} = (1-\eta_{Lm0})^2 \tag{53}$$

However, for the first order case, we use

$$\begin{aligned} h_{L1} &= \frac{A_{Lm}}{A_{Lm}+x_L^T} \\ A_{Lm} &= \frac{h_{L0}}{1-h_{L0}} \frac{1}{\sqrt{B_2 B_{Lg}} + \frac{1}{B_{Lg}} + g_L^T} \end{aligned} \tag{54}$$

as using $h_{L1} = (1-\eta_{Lm1})^2$ results in erroneous asymptotic behaviour of F_L as $x_L^T \to \infty$.

Here, we interpolate at $C_{2L} = \frac{1}{2}$ as we are analysing a module and have no information about the network. However, we can also interpolate at or near an equilibrium point for the network, or at a typical operating point.

3 Simplified Degradation Model

We next simplify the model of degradation from an implicit to an explicit form, which together with SI 2 enables the model of to be written as an ODE. We split degradation into the two cases of uniform and non-uniform degradation, where we define uniform degradation as occurring when different forms of the protein have the same degradation rate. For a transcription factor (TF) in the prototypical model with uniform degradation, the degradation rates of the monomer, free and bound dimer are equal. This differs from two distinct proteins having the same degradation rate. In many cases we can assume uniform degradation of proteins in order to simplify the protein degradation term in the ODEs (e.g. dilution only). This assumption is biologically reasonable in many cases, and more generally is a useful first approximation for the non-uniform case. If non-uniform degradation is required to be modelled, then we can use equivalent approximations to those used for simplifying regulation in SI 2.

3.1 Uniform Degradation Model

The uniform degradation assumption in the mechanistic model is

$$\begin{aligned} &\text{Dilution/Degradation} \\ &X_T \xrightarrow{\beta_t} \varnothing, \quad X_{T2} \xrightarrow{\beta_{t2}} \varnothing, \quad g_T X_{T2} \xrightarrow{\beta_{tg}} g_T \\ &\text{Uniform Rates} \\ &\beta_T := \beta_t = \beta_{t2} = \beta_{tg} \end{aligned}$$

Biologically, this is a lumped mechanistic model, as the term is composed of both dilution and degradation. The dilution terms are uniform for all conditions. Uniform degradation can be represented by

$$\begin{aligned} &\text{Degradation} \\ &X_T \xrightarrow{\beta_{t,d}} \varnothing, \quad X_{T2} \xrightarrow{2\beta_{t2,d}} X_T, \quad g_T X_{T2} \xrightarrow{2\beta_{tg,d}} g_T + X_T \\ &\text{Uniform Rates} \\ &\beta_T := \beta_{t,d} = \beta_{t2,d} = \beta_{tg,d} \end{aligned}$$

The doubled kinetic rate assumes that the protease has two sites to bind on the dimer X_{T2} or $g_T X_{T2}$. Equations of either form lead to the same reduced, deterministic equations, and so we lump the degradation and dilution biochemical equations.

Assuming uniform degradation of proteins, we can simplify the differential equation for proteins to

$$\dot{x}_T^T = k_T^{tl} m_T^T - \beta_T x_T^T$$

where β_T is a constant, rather than a function of x_T^T.

Similar to proteins, mRNA degradation can be treated as uniform as a first approximation, where the degradation rate is the same with or without a bound ribosome. Using the approximation of uniform mRNA degradation simplifies the degradation rate of mRNA to a constant $\gamma_T = \gamma_t = \gamma_{tR}$, which is otherwise dependent on the often time-varying ribosome concentration.

Mathematically, uniform degradation is similar to and thus named after uniform damping approximations used in power systems and oscillator networks [2].

3.2 Non-Uniform Degradation Model

If non-uniform degradation is required to be modelled, then we can use the approximation of η_{Tm} found in an identical fashion to η_{Lm} and R_T (see SI 2), where we have

$$\beta_T(x_T^T) = \beta_t + (\beta_{t2} - \beta_t)\eta_{Tm} + (\beta_{tg} - \beta_{t2})\frac{2g_T^T(1 - R_{T0}(\eta_{Tm}x_T^T))}{x_T^T} \tag{55}$$

We can also use interpolation of the degradation rate directly, where for a zeroth order Padé approximation with interpolation at $C_{2T} = 1/2g_T^T$, we have

$$\beta_T = \beta_t + (\beta_{t2} - \beta_t)\frac{\frac{2}{B_{Tg}} + g_T^T}{\frac{1}{\sqrt{B_{Tg}B_{T2}}} + \frac{2}{B_{Tg}} + g_T^T} + (\beta_{tg} - \beta_{t2})\frac{g_T^T}{\frac{1}{\sqrt{B_{Tg}B_{T2}}} + \frac{2}{B_{Tg}} + g_T^T} \tag{56}$$

We can similarly use interpolation to find higher order approximations (see SI 2).

4 Model Reduction of Activator

We next find the reduced model for an activator, in a similar manner to the repressor. For the case of activation, the transcription reactions in the full mechanistic model need to be replaced by the following biochemical equation:

Transcription (activator)

$$g_L X_{L2} + P \underset{\alpha_{-1}}{\overset{\alpha_1}{\rightleftharpoons}} g_L X_{L2} P \overset{\alpha_2}{\to} g_L X_{L2} + P + m_T$$

Degradation (Activation)

$$g_T X_{T2} P \overset{\beta_{tg}}{\to} g_T + P$$

Operator Binding

$$g_L + X_{L2} \underset{\alpha_{-8}}{\overset{\alpha_8}{\rightleftharpoons}} g_L X_{L2}, \quad g_L X_{L2} P \overset{\alpha_{-8}}{\to} g_L + X_{L2} + P$$

where for simplicity we assume uniform transcription factor-operator dissociation and degradation, with or without bound polymerase, noting that this assumption is generalisable.

We use equivalent derivation and nondimensionalisation as for the repressor, only showing the nondimensionalisation for the altered equations.

We set the summed variables for the transcription factor

$$\begin{aligned} x_T^T &= x_T^T + 2X_{T2} + 2C_{2T} + 2C_{1T} \\ z_T^T &= 2X_{T2} + 2C_{2T} + 2C_{1T} \\ y_T^T &= C_{2T} + C_{1T} \end{aligned} \tag{57}$$

Thus the slow variables are

$$\begin{aligned} \dot{m}_T^T &= \alpha_2 C_{1L} - \gamma_t m_T^T - (\gamma_{tR} - \gamma_t) C_{3T} \\ \dot{x}_T^T &= w_5 C_{3T} - \beta_t x_T^T - (\beta_{t2} - \beta_t) z_T^T - 2(\beta_{tg} - \beta_{t2}) y_T^T \end{aligned} \tag{58}$$

and the quasi-steady state approximations together with the conserved quantities are

$$\begin{aligned} g_L + C_{1L} + C_{2L} &= g_L^T \\ C_{1L} + C_{2L} &= y_L^T \\ \alpha_6 X_L^2 &= (\alpha_{-6} + \beta_{L2}) X_{L2} \\ \alpha_8 g_L X_{L2} &= (\alpha_{-8} + \beta_{Lg}) y_L^T \\ \alpha_1 P C_{2L} &= (\alpha_{-1} + \alpha_2 + \beta_{Lg}) C_{1L} \\ w_4 R m_T^T &= (w_4 R + w_{-4} + w_5 + \gamma_{tR}) C_{3T} \end{aligned}$$

Solving, we have the reduced differential-algebraic equations

$$\begin{aligned} \dot{m}_T^T &= g_L^T V_T^{tx} F_L - \gamma_T m_T^T \\ \dot{x}_T^T &= k_T^{tl} m_T^T - \beta_T x_T^T \\ F_L &= \frac{y_L^T}{g_L^T} \\ \beta_T &= \beta_t - (\beta_{t2} - \beta_t) \frac{z_T(y_T^T)}{x_T^T} - (\beta_{tg} - \beta_{t2}) \frac{2y_T^T}{x_T^T} \\ x_L^T &= \theta_{L1}(y_L^T) := \frac{\sqrt{y_L^T}}{\sqrt{B_{L2} B_{Lg}(g_L^T - y_L^T)}} + \frac{2y_L^T}{B_{Lg}(g_L^T - y_L^T)} + 2y_L^T \\ x_T^T &= \theta_{T1}(y_T^T) := \frac{\sqrt{y_T^T}}{\sqrt{B_{T2} B_{Tg}(g_T^T - y_T^T)}} + \frac{2y_T^T}{B_{Tg}(g_T^T - y_T^T)} + 2y_T^T \end{aligned} \tag{59}$$

where $z_T(y_T^T) = \frac{2y_T^T}{B_{Tg}(g_T^T - y_T^T)} + 2y_T^T$ is a function of y_T and where

$$\begin{aligned}
k_T^{tl} &= \frac{w_5 w_4 R}{w_{-4} + w_5 + w_4 R + \gamma_{tR}} \\
V_T^{tx} &= \frac{\alpha_2 \alpha_1 P}{\alpha_{-1} + \alpha_1 P + \alpha_2 + \beta_{Lg}} \\
B_{Lg} &= \frac{\alpha_8}{\alpha_{-8} + \beta_{lg}}, \quad B_{L2} = \frac{\alpha_6}{\alpha_{-6} + \beta_{l2}} \\
B_{Tg} &= \frac{w_8}{w_{-8} + \beta_{tg}}, \quad B_{T2} = \frac{w_6}{w_{-6} + \beta_{t2}} \\
\gamma_T &= \gamma_t + \frac{(\gamma_{tR} - \gamma_t) w_4 R}{(w_4 R + w_{-4} + w_5)}
\end{aligned} \tag{60}$$

such that V_T^{tx} is the transcription rate per non-repressed promoter, B_{L2}, B_{T2} are the effective dimerisation association constants, and B_{Lg}, B_{Tg} are the effective dimer-operator association constants. We can further simplify the parameters B_{L2}, B_{T2} and B_{Lg}, B_{Tg} by removing β_{lg} and β_{l2} if required, as per the case of the repressor (SI 1).

The model can be further simplified in an identical fashion to the repressor case in order to derive an ODE (SI 2 and 3).

Assumptions

To determine the conditions under which the reduced model holds, we again need to apply singular perturbation theory. We rewrite the differential equations which are altered from the repressor case

$$\begin{aligned}
\dot{g}_T &= -w_8 g_T X_{T2} + w_{-8} C_{2T} + w_{-8} C_{1T} + \beta_{gt} C_{2T} + \beta_{tg} C_{1T} \\
\dot{C}_{1T} &= w_1 P C_{2T} - w_{-8} C_{1T} - (w_{-1} + w_2 + \beta_{tg}) C_{1T} \\
\dot{C}_{2T} &= w_8 g_T X_{T2} - w_{-8} C_{2T} - w_1 P C_{2T} + (w_{-1} + w_2 + \beta_{tg}) C_{1T}
\end{aligned} \tag{61}$$

For the activator, we have the transformed differential equations

$$\begin{aligned}
\dot{C}_{1T} &= w_1 P y_T^T - (w_1 P + w_{-1} + w_2 + \beta_{tg}) C_{1T} \\
\dot{y}_T^T &= w_8 \left(\frac{1}{2} z_T^T - y_T^T \right) \left(g_T^T - y_T^T \right) - (w_{-8} + \beta_{tg}) y_T^T
\end{aligned} \tag{62}$$

which can be rewritten

$$\dot{y}_T^T = \frac{1}{2} w_8 g_T^T z_T^T - \left(\frac{1}{2} w_8 (z_T^T - y_T^T) + w_8 g_T^T + w_{-8} + \beta_{tg} \right) y_T^T \tag{63}$$

We have the nondimensionalisation

$$\begin{aligned}
Y_{Tn}^T &= \theta_{L1}^{-1}(X_L^n) \\
C_{1Tn} &= \frac{w_1 P}{w_1 P + w_{-1} + w_2 + \beta_{tg}} Y_{2Tn}
\end{aligned} \tag{64}$$

which results in

$$\begin{aligned}
\varepsilon_{w2} \dot{\bar{C}}_{1T} =& \bar{y}_T^T - \bar{C}_{1T} \\
\varepsilon_{w4} \dot{\bar{y}}_T =& A_{8T} \bar{z}_T^T - \left(A_{9T} \bar{z}_T^T - \frac{1}{2} A_{10T} \bar{y}_T^T + 1 - A_{9T} + A_{10T} \right) \bar{y}_T
\end{aligned} \tag{65}$$

where

$$
\begin{aligned}
\beta_{C1T} &= w_1 P_n + w_{-1} + w_2 + \beta_{tg} \\
\beta_{yT} &= \frac{1}{2} w_8 (Z_{Tn}^T - Y_{Tn}^T) + w_8 (g_T^T - Y_{Tn}^T) + w_{-8} + \beta_{tg} \\
&= w_8 X_{T2n} + w_8 g_{Tn} + w_{-8} + \beta_{tg} \\
\epsilon_{w2} &= \frac{\beta}{\beta_{C1T}}, \quad \epsilon_{w4} = \frac{\beta}{\beta_{yT}} \\
A_{8T} &= 1 + \frac{w_8 Y_{Tn}^T}{\beta_{yT}}, \quad A_{9T} = \frac{\frac{w_8}{2} Z_{Tn}^T}{\beta_{yT}}, \quad A_{10T} = \frac{w_8 Y_{Tn}^T}{\beta_{yT}}
\end{aligned}
\tag{66}
$$

where $g_{Tn} = g_T^T - Y_{Tn}^T$ and $X_{T2n} = \frac{1}{2}(Z_{Tn}^T - Y_{Tn}^T)$.

Using a similar singular perturbation form for the activator, we have the conditions

$$
\begin{aligned}
\beta_{Tn}, \gamma_{Tn} \ll &(w_4 R_n + w_{-4} + w_5 + \gamma_{tR}), (w_1 P_n + w_{-1} + w_2 + \beta_{tg}), \\
&(w_8 X_{T2n} + w_8 g_{Tn} + w_{-8} + \beta_{tg}), (4 w_6 X_{Tn} + w_{-6} + \beta_{t2})
\end{aligned}
\tag{67}
$$

as well as

$$
\beta_{tg} \eta_{To} \ll 4 w_6 X_{Tn} + w_{-6} + \beta_{t2} \tag{68}
$$

and similarly to the repressor case, we need (22). We also need to assume that if R and P are time-varying, then they are slowly time varying, as per (24).

For the case of the activator we do not need $\beta_{C2T} \ll \beta_{C1T}$, as the dynamics of C_{1T} have no effect on the dynamics of C_{2T}. Biologically, this is due to the lack of competition between polymerase and transcription factor for DNA binding sites.

5 Model Reduction - Protein Only

In this section, we determine the reduced model and assumptions for the case that only protein is modelled. We can remove the need to model mRNA if protein degradation/dilution is much smaller than mRNA degradation. For this case, the reduced biochemical equations are

$$
g_L^T \xrightarrow{k_L(x_L^T)} g_L^T + x_T^T, \; x_L^T \xrightarrow{\beta_T} \emptyset \tag{69}
$$

where k_L are the expression rates per gene, which is $k_L(x_L^T) = \frac{k_L^{tl}}{\beta_L} V_L^{tx} F_L$.

We can determine conditions for this model to hold be setting $\beta = \beta_{Tn}$ and $\varepsilon_{w7} = \frac{\beta_{Tn}}{\gamma_{Tn}}$ in (15), which leads to the alternative mRNA equation

$$
\varepsilon_{w7} \dot{m}_T^T = V_T^{tx}(g_L^T - C_{2L}) - \gamma_T m_T^T
$$

We can therefore replace (20) with the assumptions

$$
\begin{aligned}
\beta_{Tn} \ll &\gamma_{Tn}, (w_4 R_n + w_{-4} + w_5 + \gamma_{tR}), (w_1 P_n + w_{-1} + w_2), \\
&(w_8 X_{T2n} + w_8 g_{Tn} + w_{-8} + \beta_{tg}), (4 w_6 X_{Tn} + w_{-6} + \beta_{t2})
\end{aligned}
\tag{70}
$$

as well as

$$
\beta_{tg} \eta_{To} \ll 4 w_6 X_{Tn} + w_{-6} + \beta_{t2} \tag{71}
$$

and $\beta_{C2T} \ll \beta_{C1T}$ together with

$$
\beta_{Tn} \ll \frac{\beta_{C2T} \beta_{ZT} - w_8 g_{Tn}(w_{-6} + \beta_{t2})}{\beta_{C2T} + \beta_{ZT}} \tag{72}
$$

6 Model Reduction - Basal Expression

In this section, we find the reduced model for gene regulation when basal expression is included. For basal expression, we introduce the extra biochemical equations

Basal Transcription (Repression)

$$g_L X_{L2} + P \underset{\alpha_{-1r}}{\overset{\alpha_{1r}}{\rightleftharpoons}} g_L P X_{L2} \overset{\alpha_{2r}}{\rightarrow} g_L X_{L2} + P + m_T$$

$$g_L P X_{L2} \overset{\beta_{gPL}}{\rightarrow} \varnothing$$

This biochemical reaction is a lumped reaction representing the different effects such that X_{L2} bound to g_L is only partially effective in repression.

For this case, we represent the modified transcription rate in (6) as

$$k_T^{tx} = V_{T,rep}^{tx} + (V_T^{tx} - V_{T,rep}^{tx})F_L$$

where

$$V_{T,rep}^{tx} = \frac{\alpha_{2r}\alpha_{1r}P}{\alpha_{-1r} + \alpha_{1r}P + \alpha_{2r}}$$

The assumptions for the singular perturbation can be found in an equivalent manner to SI 1, while TF degradation rate in the reduced models can be found in a similar manner to that described in SI 3.

7 Model Reduction - Inducers

In this section, we include a prototypical mechanistic model of the effect of inducers on the transcription factors. We use a relatively simple biochemical model of inducer binding to illustrate the methodology, noting that more complicated biochemical models can also be used if required. We include the extra biochemical reactions

Inducer

$$X_L + I \underset{k_{-I1}}{\overset{k_{I1}}{\rightleftharpoons}} X_L I$$

$$X_{L2} + 2I \underset{k_{-I2}}{\overset{k_{I2}}{\rightleftharpoons}} X_{L2} 2I$$

$$g_L X_{L2} + 2I \overset{k_{I2}}{\rightarrow} X_{L2} 2I + g_L$$

Degradation

$$X_L I \overset{\beta_I}{\rightarrow} I$$

$$X_{L2} 2I \overset{\beta_{I2}}{\rightarrow} 2I$$

in Equation (1) in the paper. We have the total protein concentration

$$x_L^T = X_L + C_I + 2X_{L2} + 2C_{I2} + 2C_{2L} \tag{73}$$

where $C_I = [X_L I]$ and $C_{2I} = [X_{L2} 2I]$. The quasi-steady state approximations are

$$\begin{aligned} B_{L2} X_L^2 &= X_{L2} \\ X_{L2} &= \frac{C_{2L}}{B_{Lg}(g_L^T - C_{2L})} \\ C_I &= B_I I X_L \\ C_{I2} &= B_{I2} I^2 (X_{L2} + C_{2L}) \end{aligned} \tag{74}$$

where

$$\begin{aligned} B_{Lg} &= \frac{\alpha_8}{\alpha_{-8} + \beta_{lg} + k_{I2} I^2}(1 - A_{1L}) \\ B_I &= \frac{k_I}{k_{-I} + \beta_I}, \quad B_{I2} = \frac{k_{I2}}{k_{-I2} + \beta_{I2}} \end{aligned} \tag{75}$$

See SI 1 for further information on A_{1L} and B_{L2}. Thus

$$\begin{aligned} x_L^T =& (1 + B_I I) X_L + 2(1 + B_{I2} I^2) X_{L2} + 2(1 + B_{I2} I^2) C_{2L} \\ =& \frac{(1 + B_I I)}{\sqrt{B_{L2} B_{Lg}}} \frac{\sqrt{C_{2L}}}{\sqrt{(g_L^T - C_{2L})}} + \frac{2(1 + B_{I2} I^2) C_{2L}}{B_{Lg}(g_L^T - C_{2L})} + 2(1 + B_{I2} I^2) C_{2L} \end{aligned} \tag{76}$$

We assume that the free inducer concentration is a constant. However, the non-constant inducer case can be treated using Padé approximations as per the methodology in SI 2.

Now (76) has the same form as the non-inducer case, but with altered parameters. Thus we have

$$\begin{aligned} F_L &= \begin{cases} \sqrt{W^2 + \frac{1}{B_{Lg} g_L^T}} - W & \varepsilon_L \leq 1 \\ \frac{1}{1 + \frac{1}{(1+B_I I)^2} B_{L2} B_{Lg} h_L {x_L^T}^2} & \varepsilon_L > 1 \end{cases} \\ W &= \frac{\eta_{Lm} x_L^T}{4(1 + B_{I2} I^2) g_L^T} + \frac{1}{2 B_{Lg} g_L^T} - \frac{1}{2} \\ \varepsilon_L &= \frac{(1 + B_I I)\sqrt{B_{Lg}}}{2(1 + B_{I2} I^2)(1 + B_{Lg} g_L^T)\sqrt{B_{L2}}} \end{aligned} \tag{77}$$

For the multimer dominant case, we have

$$\begin{aligned} \eta_{L0} &= 1 \\ \eta_{L1} &= \frac{x_L^T}{x_L^T + \frac{(1+B_I I)}{\sqrt{B_{L2} B_{Lg}}} \sqrt{F_{L0} \left(x_L^T\right)^{-1} - 1}} \end{aligned} \tag{78}$$

For the monomer dominant case, we have

$$\begin{aligned} h_{L0} &= 1 \\ h_{L1} &= \frac{x_L^T}{x_L^T + 4(1 + B_{I2} I^2)\left(\frac{1}{B_{Lg}}(F_{L0}^{-1} - 1) + g_L^T(1 - F_{L0}(x_L^T))\right)} \end{aligned} \tag{79}$$

The degradation term for the model can be determined using a similar methodology to SI 3. The assumptions for the singular perturbation can be found in an equivalent manner to SI 1.

8 Model Reduction - Unbound Dimer Only

In this section, we find a simplified version of the regulation term for the multimer dominant case. We start with the proposed model's multimer dominant regulation term

$$F_L = \sqrt{\left(\frac{\eta_{Lm} x_L^T}{4 g_L^T} + \frac{1}{2 B_{Lg} g_L^T} - \frac{1}{2}\right)^2 + \frac{1}{B_{Lg} g_L^T}} - \left(\frac{\eta_{Lm} x_L^T}{4 g_L^T} + \frac{1}{2 B_{Lg} g_L^T} - \frac{1}{2}\right) \tag{80}$$

and assume that $g_L^T \ll \frac{1}{B_{Lg}}$, for which the system is predominantly unbound dimer for all concentrations of x_L^T, ignoring monomer by using the previous assumptions. Using $\frac{1}{B_{Lg} g_L^T} \gg 1$, then

$$\left(\frac{\eta_{Lm} x_L^T}{4 g_L^T} + \frac{1}{2 B_{Lg} g_L^T} - \frac{1}{2}\right)^2 \gg \frac{1}{B_{Lg} g_L^T} \tag{81}$$

and so we have

$$\begin{aligned} F_L &= \left|\left(\frac{\eta_{Lm} x_L^T}{4 g_L^T} + \frac{1}{2 B_{Lg} g_L^T} - \frac{1}{2}\right)\right| \left(\sqrt{1 + \frac{\frac{1}{B_{Lg} g_L^T}}{\left(\frac{\eta_{Lm} x_L^T}{4 g_L^T} + \frac{1}{2 B_{Lg} g_L^T} - \frac{1}{2}\right)^2}} - 1\right) \\ &= \left|\frac{\eta_{Lm} x_L^T}{4 g_L^T} + \frac{1}{2 B_{Lg} g_L^T} - \frac{1}{2}\right| \left(\frac{\frac{1}{B_{Lg} g_L^T}}{2\left(\frac{\eta_{Lm} x_L^T}{4 g_L^T} + \frac{1}{2 B_{Lg} g_L^T} - \frac{1}{2}\right)^2}\right) \\ &= \frac{1}{1 + \frac{B \eta_{Lm}}{2} x_L^T} \end{aligned} \tag{82}$$

9 Model Reduction - Multiple Operators

In this section, we determine reduced models of gene regulation when additional operators are included. The effect of additional operators can be important for the multimer dominant case of regulation, while for the monomer dominant case, the effect of adding additional operators is typically smaller. To incorporate the additional operators in the reduced models, we can once again split the model into multiple cases and use perturbation theory. For the multimer dominant case, we initially analyse the three special cases of extra operators with much higher, equal and much weaker affinity, when compared to the primary operator. We then use Padé approximations to describe regulation functions for cases in between the three asymptotic cases. Here we use perturbation theory to determine Padé approximations, although we can alternatively use interpolation. The extra operator has no affect on the monomer dominant case for the initial approximation, but is included in higher order approximations through the multimerisation efficiency.

For the case of a second operator, we have the added biochemical equation

$$O_L + X_{L2} \underset{\alpha_{-9}}{\overset{\alpha_9}{\rightleftharpoons}} X_{L2} O_L \tag{83}$$

along with relevant degradation term. Setting $C_{4L} = [X_{L2} O_L]$, we have the modified total protein equation

$$x_L^T = X_L + 2 X_{L2} + 2 C_{2L} + 2 C_{4L}$$

along with the quasi-steady state

$$C_{4L} = B_{Lo} O_L X_{L2} \tag{84}$$

where $B_{Lo} = \frac{\alpha_9}{\alpha_{-9}}$. Now we have the conserved quantity $C_{4L} + O_L = O_L^T$ and so $C_{4L} = O_L^T \frac{B_{Lo} X_{L2}}{1+B_{Lo} X_{L2}}$. Thus we have

$$x_L^T = \frac{\sqrt{C_{2L}}}{\sqrt{B_{L2} B_{Lg}(g_L^T - C_{2L})}} + \frac{2C_{2L}}{B_{Lg}(g_L^T - C_{2L})} + 2C_{2L} + 2O_L^T \frac{B_{Lo} C_{2L}}{B_{Lg}(g_L^T - C_{2L}) + B_{Lo} C_{2L}} \tag{85}$$

The perturbation problem for the dimer dominant regulation is

$$\begin{aligned}
& A_1 \bar{C}_{2L} + A_O \frac{\bar{C}_{2L}}{1 - \bar{C}_{2L} + \frac{B_{Lo}}{B_{Lg}} \bar{C}_{2L}} + (1 - A_1 - A_O) \frac{\bar{C}_{2L}}{1 - \bar{C}_{2L}} + \varepsilon_L \sqrt{\frac{\bar{C}_{2L}}{1 - \bar{C}_{2L}}} = A_2 \\
& A_1 = \frac{B_{Lg} g_L^T}{1 + B_{Lg} g_L^T + B_{Lo} O_L^T} \\
& A_O = \frac{B_{Lo} O_L^T}{1 + B_{Lg} g_L^T + B_{Lo} O_L^T} \\
& A_2 = \frac{B_{Lg} x_L^T}{2(1 + B_{Lg} g_L^T + B_{Lo} O_L^T)} \\
& \varepsilon_L = \frac{\sqrt{B_{Lg}}}{2(1 + B_{Lg} g_L^T + B_{Lo} O_L^T)\sqrt{B_{L2}}}
\end{aligned} \tag{86}$$

From this we can see that a system with extra operators is more likely to be multimer dominant. The assumptions for the singular perturbation can be found in an equivalent manner to SI 1, noting that the coupling condition equivalent to (17) requires three variables to be taken into account, rather than two.

9.1 Multimer Dominant Case

For the multimer dominant case, we analyse the three special cases of $B_{Lo} \gg B_{Lg}$, $B_{Lo} \approx B_{Lg}$ and $B_{Lo} \ll B_{Lg}$. We then use Padé approximations to represent the cases in-between these asymptotic cases, so that the reduced models can be determine for all values of B_{Lo}.

We have the total dimer concentration

$$z_L^T = \frac{2C_{2L}}{B_{Lg}(g_L^T - C_{2L})} + 2C_{2L} + 2O_L^T \frac{B_{Lo} C_{2L}}{B_{Lg}(g_L^T - C_{2L}) + B_{Lo} C_{2L}}$$

In the following, we treat the multimerisation efficiency and the effect of added operators as two separate perturbation problems. This minor informality is sufficient for the zeroth and first order approximations determined here, but more care is required for higher order terms or determining error bounds if ε_L and the small parameters in this section are of the same order of magnitude.

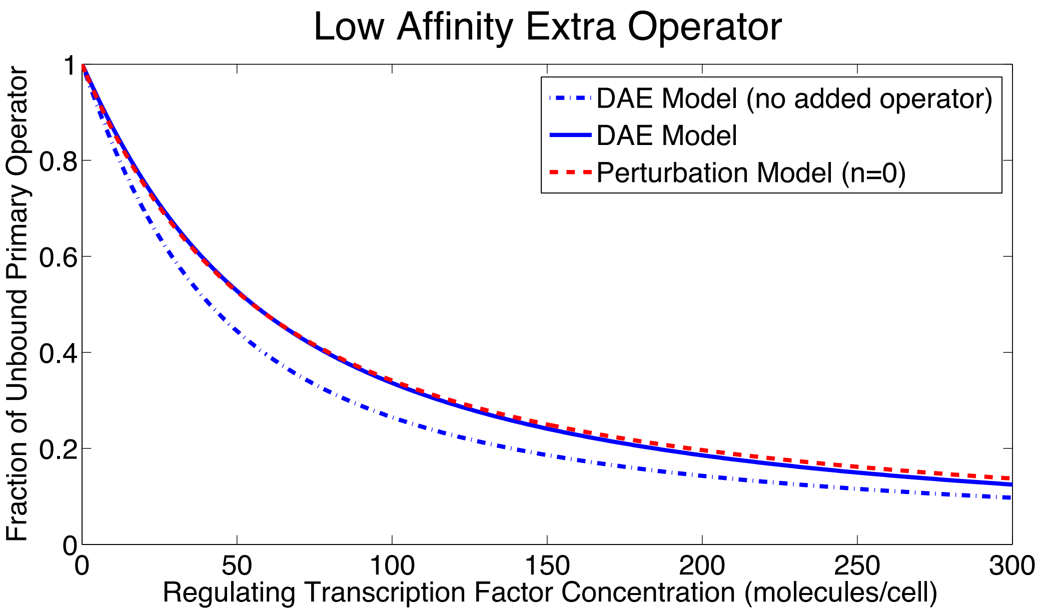

Figure S8: Comparison of DAE models with (85) and without (6) secondary operators as well as the perturbation model (87), for the case of a weak secondary operator-binding site. Parameters are $g_L^T = 10$, $O_L^T = 100$, $B_{Lg} = 0.0667$, $B_{L2} = 10$, $B_{Lo} = 0.005$.

9.1.1 Low Affinity Extra Operator

If $B_{Lo} \ll B_{Lg}$ then we use the form

$$\begin{aligned} F_L &= F_{L0}(x_L^T) = \sqrt{W_L^2 + \frac{A}{B_{Lg} g_L^T}} - W_L \\ W_L &= \frac{\eta_{Lm} x_L^T}{4 g_L^T} + \frac{A}{2 B_{Lg} g_L^T} - \frac{1}{2} \\ A &= 1 + B_{Lo} O_L^T \frac{1}{1 + \varepsilon_{Lo}(F_L^{-1} - 1)} \\ \varepsilon_{Lo} &= \frac{B_{Lo}}{B_{Lg}} \end{aligned} \tag{87}$$

For the first order approximation, we have

$$A = A_0 = 1 + B_{Lo} O_L^T \tag{88}$$

Adding more weak operators effectively weakens the binding affinity $\left(\frac{B_{Lg}}{A}\right)$ of the primary operator. An example of this is due to non-specific binding.

For a higher order approximation, using a Padé approximation, we have

$$A = A_1 = 1 + B_{Lo} g_L^T \frac{1}{1 + \frac{B_{Lo}}{B_{Lg}}(F_{L0}^{-1} - 1)} \tag{89}$$

9.1.2 Equal Affinity Extra Operator

For an equal affinity extra operator, we have

$$\begin{aligned} F_L &= \sqrt{W_L^2 + \frac{1}{B_{gL} A g_L^T}} - W_L \\ W_L &= \frac{\eta_{Lm} x_L^T}{4 A g_L^T} + \frac{1}{2 B_{gL} A g_L^T} - \frac{1}{2} \\ A &= 1 + \frac{B_{Lo} O_L^T}{B_{Lg} g_L^T} \frac{1}{1 + \varepsilon_{Lo}(1 - F_L)} \\ \varepsilon_{Lo} &= \frac{B_{Lo} - B_{Lg}}{B_{Lg}} \end{aligned} \tag{90}$$

For a zeroth order approximation, we can use

$$A_0 = 1 + \frac{B_{Lo} O_L^T}{B_{Lg} g_L^T}, \quad F_{L0} = F_L|_{A=A_0} \tag{91}$$

Thus adding more equal affinity operators effectively increases the gene copy number in the regulation term ($A g_L^T$).

For a first order approximation, we can use

$$A = A_1 = 1 + \frac{B_{Lo} O_L^T}{B_{Lg} g_L^T} \frac{1}{1 + \frac{B_{Lo} - B_{Lg}}{B_{Lg}} (1 - F_{L0})}, \quad F_{L1} = F_L|_{A=A_1} \tag{92}$$

9.1.3 High Affinity Extra Operator

If $B_{Lo} \gg B_{Lg}$ then we have

$$\begin{aligned} F_L &= \sqrt{W_L^2 + \frac{1}{B_{Lg} g_L^T}} - W_L \\ W_L &= \frac{\eta_{Lm} x_L^T - A}{4 g_L^T} + \frac{1}{2 B_{Lg} g_L^T} - \frac{1}{2} \\ A &= 2 O_L^T \frac{1}{1 + \varepsilon_{Lz} \left(\frac{F_L}{1 - F_L} \right)} \\ \varepsilon_{Lz} &= \frac{B_{Lg}}{B_{Lo}} \end{aligned} \tag{93}$$

There is a boundary layer near $C_{2L} = 0$, and we have the zeroth order outer solution

$$A = A_{0,out} = 2 O_L^T \tag{94}$$

It can be noted that a higher affinity extra operator effectively removes transcription factor. For a rough solution, we can use the estimate

$$= A_{0,out} = \begin{cases} 2 O_L^T & \text{if } x_L^T \geq 2 O_L^T \\ x_L^T & \text{otherwise} \end{cases} \tag{95}$$

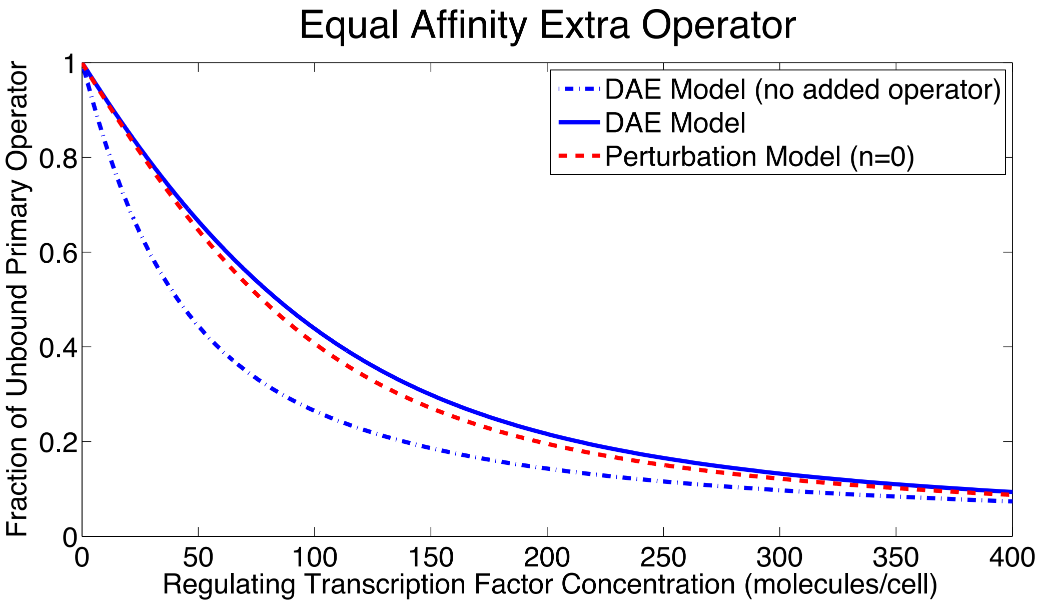

Figure S9: Comparison of DAE models with (85) and without (6) secondary operators as well as the perturbation model (90), for the case of an approximately equal secondary operator-binding site. Parameters are $g_L^T = 10$, $O_L^T = 50$, $B_{Lg} = 0.0667$, $B_{L2} = 10$, $B_{Lo} = 0.05$.

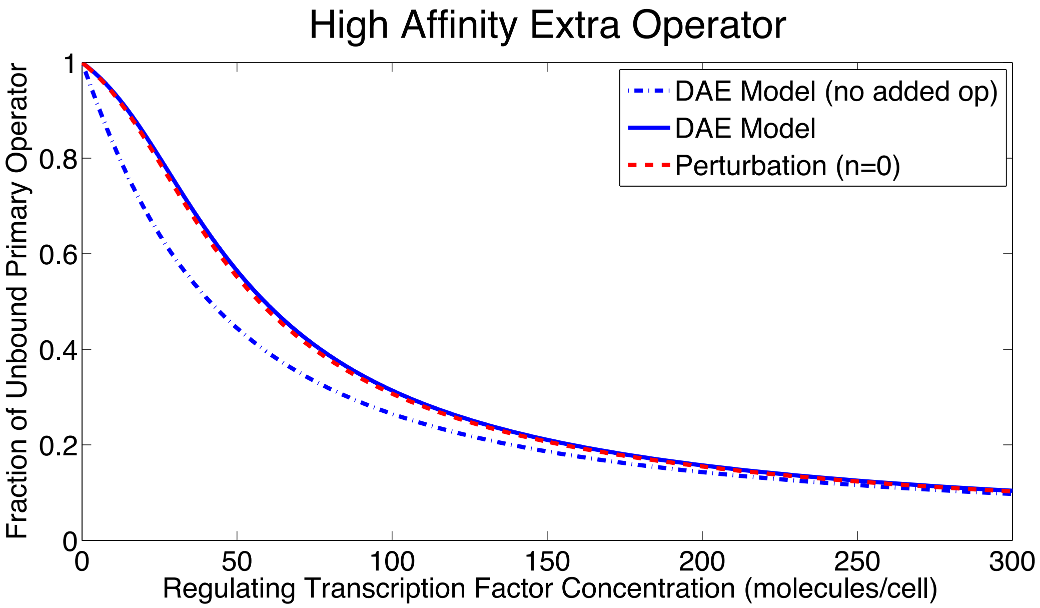

Figure S10: Comparison of DAE models with (85) and without (6) secondary operators as well as the perturbation model (93), for the case of a strong secondary operator-binding site. Parameters are $g_L^T = 10$, $O_L^T = 10$, $B_{Lg} = 0.0667$, $B_{L2} = 10$, $B_{Lo} = 0.5$.

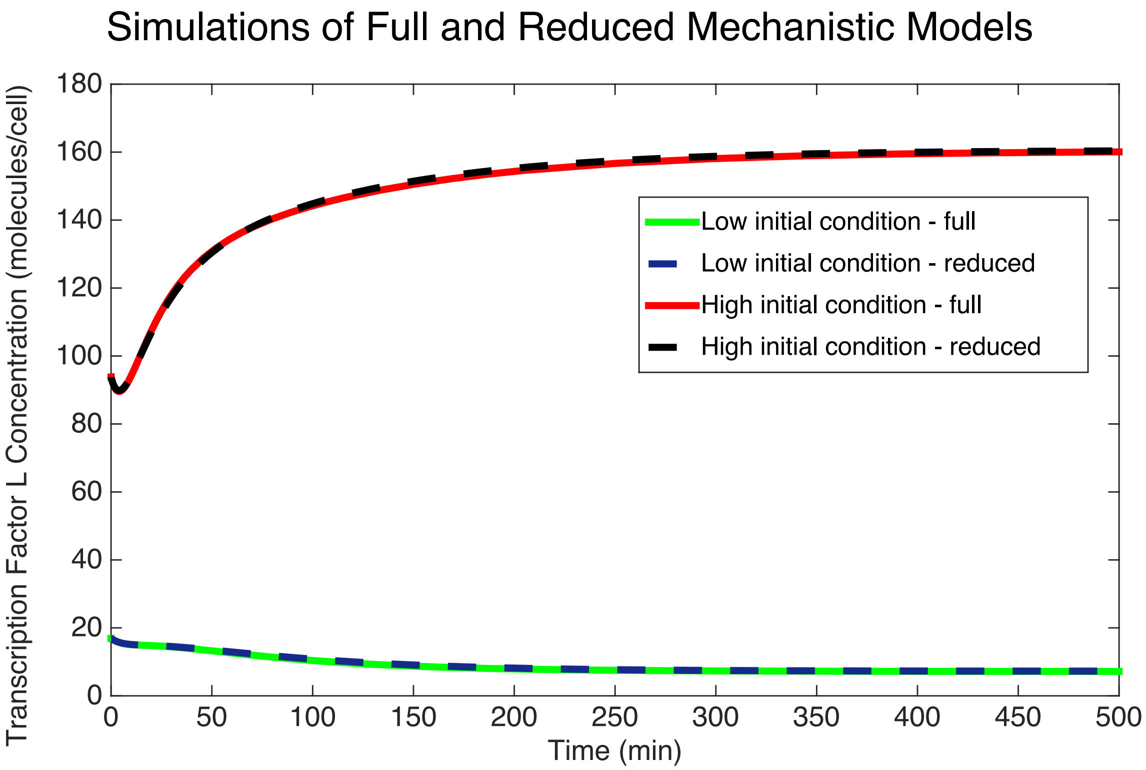

Figure S11: Simulation of full and reduced mechanistic models for a genetic toggle switch with additional TF binding sites for one TF. The TF concentration for one half of the toggle switch is shown. A close match between the full and reduced models can be seen. The parameters used match those in Figure 4 in the main section (see Materials and Methods). The kinetic rates not discussed in Materials and Methods are $P = 100, R = 100, a_4 = 0.03, a_{-4} = 0.8, a_5 = 2, a_6 = 0.5125, a_{-6} = 1, a_8 = 1.845, a_{-8} = 1, a_9 = 5.125, a_{-9} = 1, a_1 = 0.03, a_{-1} = 1, a_2 = 2$. $w_4 = 0.02, w_{-4} = 0.8, w_5 = 2, w_6 = 0.5125, w_{-6} = 1, w_8 = 0.82, w_{-8} = 1, w_1 = 0.03, w_{-1} = 1, w_2 = 2$.

by noting that saturation of A with increasing x_L^T occurs when $C_{2L} \ll g_L^T$ and so any error is relatively small.

For a more accurate estimate across a range of parameters, it is simpler to use the low affinity case to determine the dimer bound to second operator, using (87) with B_{Lo}, O_L^T swapped with B_{Lg}, g_L^T to obtain F_O. From this, we obtain

$$\begin{aligned} A &= A_0 = 2O_L^T(1 - F_{O0}) \\ A &= A_1 = 2O_L^T(1 - F_{O1}) \end{aligned} \tag{96}$$

9.2 Monomer Dominant Case

For the monomer dominant case, the regulation term is not affected by the extra operators for the zeroth order approximation. However, for higher order approximations there is an effect.

Using an equivalent methodology to the single operator case, we have

$$
\begin{aligned}
h_{L0} &= (1-\eta_{Lm0})^2 = 1 \\
h_{L1} &= (1-\eta_{Lm1})^2 = \frac{x_L^T}{x_L^T + 4g_L^T(1-F_{L0}) + \frac{4}{B_{Lg}}(F_{L0}^{-1}-1) + 4O_L^T \frac{B_{Lo}g_L^T(1-F_{L0})}{B_{Lg}g_L^T F_{L0} + B_{Lo}g_L^T(1-F_{L0})}}
\end{aligned} \tag{97}
$$

10 Regulation Efficiency

In this section, we discuss regulation efficiency, an important concept in gene regulation. We define the regulation efficiency to be the fraction of transcription factor, in monomer units, which is bound to an operator. The efficiency of regulation η_{Lg} for the transcription factor is defined here as

$$\eta_{Lg} := \frac{2g_L X_{L2}}{x_L^T} \tag{98}$$

noting the 2 is required as there are two monomer units in a dimer, and also noting that regulatory (η_{Lg}) and multimerisation (η_{Lm}) efficiency are distinct. The efficiency of regulation η_{Lg} for the dimer can be estimated by

$$\eta_{Lg} = \frac{2g_L^T(1-F_L)}{x_L^T} \tag{99}$$

where F_L is given in Equation 3 in the paper.

For the case of multiple operators, the efficiency is defined by

$$\eta_{Lg} := \frac{2g_L X_{L2} + 2X_{L2}O_L}{x_L^T} \tag{100}$$

and the efficiency can be estimated by

$$\eta_{Lg} = \frac{2g_L^T(1-F_L)}{x_L^T} + \frac{2O_L^T(1-F_O)}{x_L^T} \tag{101}$$

where F_L and F_O are the regulation functions for the respective promoters, found using Equation 3 in the main section, with relevant corrections as described in SI 9.

References

[1] U. Alon. *An Introduction to Systems Biology: Design Principles of Biological Circuits*. CRC Press, 2007.

[2] A.R. Bergen and V. Vittal. *Power Systems Analysis*. Prentice Hall, 2000.

[3] J. A. M. Borghans, R. J. De Boer, and L.A. Segel. Extending the quasi-steady state approximation by changing variables. *Bulletin of Mathematical Biology*, 58(1):43–63, 1996.

[4] J. Elf, G Li, and X. S. Xie. Probing transcription factor dynamics at the single-molecule level in a living cell. *Science*, 316:1191–1194, 2007.

[5] M. B. Elowitz and S. Leibler. A synthetic oscillatory network of transcriptional regulators. *Nature*, 403:335–338, 2000.

[6] A. Gyorgy and D. Del Vecchio. Modular composition of gene transcription networks. *PLoS Comput Biol*, 10(3):doi: 10.1371/journal.pcbi.1003486, 2014.

[7] P. Hammar, M. Wallden, D. Fange, F. Persson, O. Baltekin, G. Ullman, P. Leroy, and J. Elf. Direct measurement of transcription factor dissociation excludes a simple operator occupancy model for gene regulation. *Nature Genetics*, 46(4):405–410, 2014.

[8] S. Jayanthi, K. S. Nilgiriwala, and D. Del Vecchio. Retroactivity controls the temporal dynamics of gene transcription. *ACS Biology*, 2:431–441, 2013.

[9] H. Khalil. *Nonlinear Systems*. Prentice Hall, 2002.

[10] J.D. Murray. *Mathematical Biology I: An Introduction*. Springer, 2002.

[11] H.M. Salis, E. A. Mirsky, and C.A. Voigt. Automated design of synthetic ribosome binding sites to control protein expression. *Nature Biotechnology*, 27:946 – 950, 2009.
